# 3D Bioprinted Blood–Brain–Tumor Barrier Spheroid‐Based In Situ Evaluation of Anticancer Compounds Against Glioblastoma: ROS‐Producing Ruthenium as a Potential Therapeutic Candidate

**DOI:** 10.1002/advs.78150

**Published:** 2026-10-06

**Authors:** Hyunjung Lee, Chan Ho Jeon, Doyun Kim, Minjae Lee, Minji Kwon, Joon Myong Song

**Affiliations:** ^1^ College of Pharmacy Seoul National University Seoul Republic of Korea

**Keywords:** 3D bioprinting, blood‐brain tumor barrier (BBTB), glioblastoma (GBM), in situ anticancer efficacy evaluation, tumor spheroid imaging

## Abstract

The blood–brain–tumor barrier (BBTB) penetration by anticancer drugs and their effects on glioblastoma (GBM) are assessed using 3D bioprinted‐spheroids. Comparative analysis reveals prominent cytoplasmic mixing between pericytes and the human GBM cell line U‐87MG, expressions of tight junction proteins ZO‐1, occludin, and claudin‐5, which act as a barrier to the paracellular transport across BBTB, and the expression of ABC transporter such as P‐glycoprotein (P‐gp) in the vascular endothelial cell layer of BBTB spheroids. Anticancer drug‐induced U‐87 MG cell death is quantified through in situ imaging of spheroids using a cancer cell death probe. Paclitaxel and cisplatin, known P‐gp substrates, do not demonstrate transcellular transport in BBTB spheroids, whereas temozolomide and a ruthenium (Ru) compound induce marked U‐87 MG cell death. Relative BBTB permeability is calculated as a function of IC_50_ and treatment concentration. The Ru compound, a partial P‐gp substrate, exhibits greater anticancer efficacy compared with temozolomide, which is not a P‐gp substrate, in BBTB spheroids. The Ru compound attenuates tight junction protein expression (occludin and ZO‐1) in vascular endothelial cells through the released reactive oxygen species (ROS), increases ECM degradation via elevated matrix metalloproteinase‐9 in vascular endothelial and astrocytes, and promotes expression of the vasoconstrictor endothelin‐1.

## Graphical abstract

The blood–brain–tumor barrier (BBTB) penetration by anticancer drugs and their effects on glioblastoma (GBM) is assessed using 3D bioprinted‐spheroids. Anticancer drug‐induced U‐87 MG cell death is quantified through in situ imaging of spheroids using a cancer cell death probe. The Ru compound, a partial P‐gp substrate, induces greater tumor‐cell death than temozolomide, a non P‐gp substrate, in BBTB spheroids.

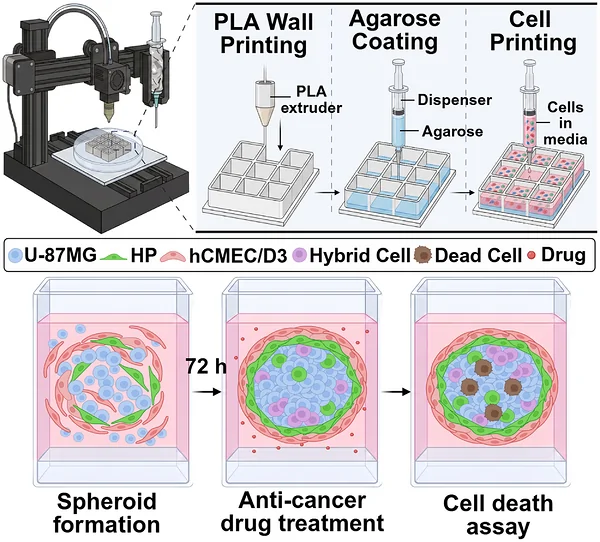

## 1 Introduction

Generally, tumor studies employ a subcutaneous model in which cancer cells are inoculated into the space beneath the skin of mice or rats to form tumors. However, since glioblastoma (GBM) develops in vivo within the brain, it grows in a unique microenvironment characterized by the brain's specialized extracellular matrix (ECM), structural features, and immune milieu mediated by resident immune cells such as microglia [1]. The subcutaneous model presents challenges in replicating authentic tumor characteristics and drug responses as it does not mimic the brain‐specific microenvironment [2, 3]. Moreover, drug delivery to brain tumors is restricted by the blood–brain barrier (BBB); however, this barrier is absent in subcutaneous models, thereby limiting their ability to recapitulate the drug delivery challenges encountered in clinical GBM [4]. Therefore, researchers employ orthotopic models for GBM investigations, which offer greater predictive therapeutic value but require complex surgical and technical expertise [5]. In orthotopic models, transplanting tumor cells into the brain through a skull incision requires precise surgical skills, as procedural variability may compromise the study results due to tissue damage and inflammatory reactions during the procedure. Monitoring the progress of tumor growth is equally demanding for tumors located deep within the brain. Unlike the subcutaneous model, which can be assessed with the naked eye, the orthotopic model requires the use of advanced imaging modalities, such as magnetic resonance imaging (MRI) and computed tomography (CT), which require specialized expertise, or alternatively relies on indirect monitoring through body weight measurements [6]. Furthermore, animal models often fail to fully recapitulate human tissue characteristics and are subject to rigorous ethical constraints. Hence, the need for in vitro mimic models, as well as in vivo models such as subcutaneous and orthotopic models, is increasing in preclinical studies. In vitro tumor‐mimicking models are increasingly recognized for their ability to replicate actual organs and tissues, enabling researchers to test, screen, and evaluate drug candidates during the preclinical stage [7, 8, 9]. Furthermore, prospects for animal‐free in vitro models have improved considerably following recent Food and Drug Administration approval of alternative methods for preclinical trials.

A BBB mimic model in which constituent BBB cells are co‐cultured within a hydrogel has been reported [10]. This system demonstrates marked increases in the expression of tight junction (TJ) proteins, such as ZO‐1 and claudin‐5, compared with a two‐dimensional (2D) model, and enables the self‐organization of constituent cells into microvasculature‐like structures within the gel. Additionally, BBB models constructed within microfluidic systems have been reported. The microfluidic flow within the chip simulates the shear stress induced by blood flow in vivo, influencing the maturation of vascular cells. Typically, endothelial cells and pericyte/astrocyte compartments are separated by a membrane layer incorporated into the microfluidic chip, facilitating convenient assessment of BBB permeability of test molecules [11]. Recently, blood–brain–tumor barrier (BBTB) mimic models have garnered substantial attention. Three‐dimensional (3D) printing using IP‐S photoresist has been employed to fabricate tubular scaffolds with 4 µm pores. Endothelial cells and astrocytes are seeded on the inner and outer surfaces of the scaffold, respectively, whereas tumor spheroids comprised of the human GBM cell line U‐87 MG are grown on a separate magnetic scaffold and introduced to the assembly. This model is utilized to evaluate anticancer agent‐induced cell death in GBM tumors. Conventionally, quantifying GBM cell death via cell viability/death probe molecules is challenging due to their limited BBB permeability; however, this model allows probes to act directly on tumor cells without traversing the BBB, as the BBB and tumor compartments are distinctly separated. However, the compartmentalizing scaffold may impede direct cellular interactions among BBB constituent cells.

Emerging studies have reported notable phenomena arising from interactions between pericytes and GBM [12, 13, 14]. In vivo, GBM tumors often promote tumor progression through direct interactions with pericytes. Furthermore, GBM cells can cause cytoplasmic mixing between pericytes and tumor cells. Additionally, these interactions may induce changes in the cellular arrangement of BBB constituents, potentially impacting BBB permeability [15, 16]. Previous BBTB models facilitated only indirect interactions due to scaffold separation and were inadequate to support direct interactions, such as cytoplasmic mixing.

Seo et al. developed a perfusable three‐dimensional BBB–GBM microfluidic model by forming endothelialized cylindrical channels within a type I collagen hydrogel containing human astrocytes and brain vascular pericytes and subsequently incorporating T98G or U87MG spheroids adjacent to the matured BBB. This platform enabled investigation of reciprocal BBB–GBM interactions, tumor‐induced vascular changes, and proof‐of‐concept assessment of barrier‐associated drug transport and tumor response [17]. Separately, Cho et al. established self‐assembled BBB spheroids composed of endothelial cells, pericytes, and astrocytes that reproduced tight‐junction and P‐gp functions and enabled screening of BBB‐penetrating compounds [18]. Because these BBB spheroids did not contain tumor cells, barrier penetration and tumor‐cell responses could not be evaluated concurrently within the same intact construct. A tumor‐containing BBTB spheroid linking compound transport across the barrier to spatially resolved tumor‐cell responses would therefore complement these platforms.

In this study, we introduce a brain tumor‐mimicking BBTB spheroid that enables in situ measurement of BBTB permeability to anticancer agents and the subsequent induction of cancer cell death at the single‐cell level within intact spheroids, without dissociating them into individual GBM cells. The self‐organized spheroids were fabricated using a 3D bioprinting system, facilitating direct interactions hCMEC/D3 endothelial cells, HBVP pericytes, and U‐87 MG glioblastoma cells. This model demonstrated robust expression of TJ proteins and ATP‐binding cassette (ABC) transporters, including P‐gp, which are essential for establishing a functional BBTB. Using this platform, the penetration, intracellular accumulation and antitumor efficacy of a synthetic Ru compound against GBM were evaluated in situ, providing an initial assessment of its potential as a therapeutic candidate for brain tumors.

Ruthenium (Ru) compounds exert anticancer effects by generating reactive oxygen species (ROS), such as singlet oxygen (^1^O_2_) and hydroxyl radicals (•OH), which are typically byproducts of intracellular metabolism. Due to their rapid proliferation and elevated metabolic rates, cancer cells sustain higher ROS levels than normal cells. Notably, cancer cells survive these high ROS levels by relying on the antioxidant system [19, 20]. An additional increase in ROS, induced by the uptake of ROS‐generating agents such as the Ru compound, heightens oxidative stress, damaging DNA, proteins, and lipids within the cancer cells, ultimately resulting in their death [19, 21]. Recent studies have actively examined the correlation between ROS and BBB integrity. While ROS participate in normal cell signaling, excessive production can compromise/alter TJ proteins in vascular endothelial cells, increasing BBB permeability [20, 22]. Based on these properties, we designed an ROS‐producing Ru compound that downregulates TJ proteins and upregulates matrix metalloprotease‐9 (MMP‐9) and the vasoconstrictor endothelin‐1 (ET‐1), enabling it to traverse the BBTB via paracellular transport as well as transcellular transport via passive diffusion partially. Recently, cationic aggregation‐induced‐emission (AIE)‐active anticancer agents that cross the BBB have shown ROS‐based efficacy in an orthotopic glioma model in vivo [23].

Collectively, the Ru compound (a ruthenium (II) polypyridyl complex isolated as [Ru(bpy)_2_(encip)](ClO_4_)_2_, with a molecular weight of 871.9 g mol^−^^1^) presented in this study exhibits substantial distribution into the fabricated BBTB spheroid model, and represents the first ruthenium‐based ROS‐generating agent evaluated in the constructed system [24]. Additionally, this study presents the model as an alternative tool for the preliminary assessment of compound distribution and tumor‐cell cytotoxicity through in situ evaluation of cancer cell death at the single‐cell level, so that the constructed spheroid platform complements conventional permeability measurements.

## 2 Results

### 2.1 Cellular Arrangement of BBTB Spheroid

To investigate the spontaneous formation of spheroids, HBVPs, hCMEC/D3 endothelial cells, and U‐87 MG glioblastoma cells were co‐cultured in a 3D‐printed, agarose‐coated microwell platform (Figure 1). Throughout this study, BBTB spheroids were assembled from hCMEC/D3 cells, HBVPs and U‐87 MG cells at a 1:1:1 ratio, whereas BBB spheroids, used as the tumor‐free comparator, comprised hCMEC/D3 cells, primary human astrocytes and HBVPs at the same ratio. Prior to spheroid assembly, each cell type was individually labeled with a unique CellTracker dye to facilitate spatial analysis within the assembled spheroids. Cells were examined every 24 h for 5 days under brightfield microscopy (Figure 2A), revealing small spheroid formation starting from Day 1 and progressive compaction thereafter. By Day 3, spheroids reached an average diameter of approximately 300 µm; these Day‐3 spheroids were used in all subsequent experiments. Spatial analysis of day‐3 spheroids identified hCMEC/D3 endothelial cells in the outermost layer, HBVPs forming an intermediate layer, and U‐87 MG cells comprising the core (Figure 2B–D) [18]. Notably, a substantial proportion of HBVPs co‐localized within the U‐87 MG core region (Figure 2C,D).

**FIGURE 1:**
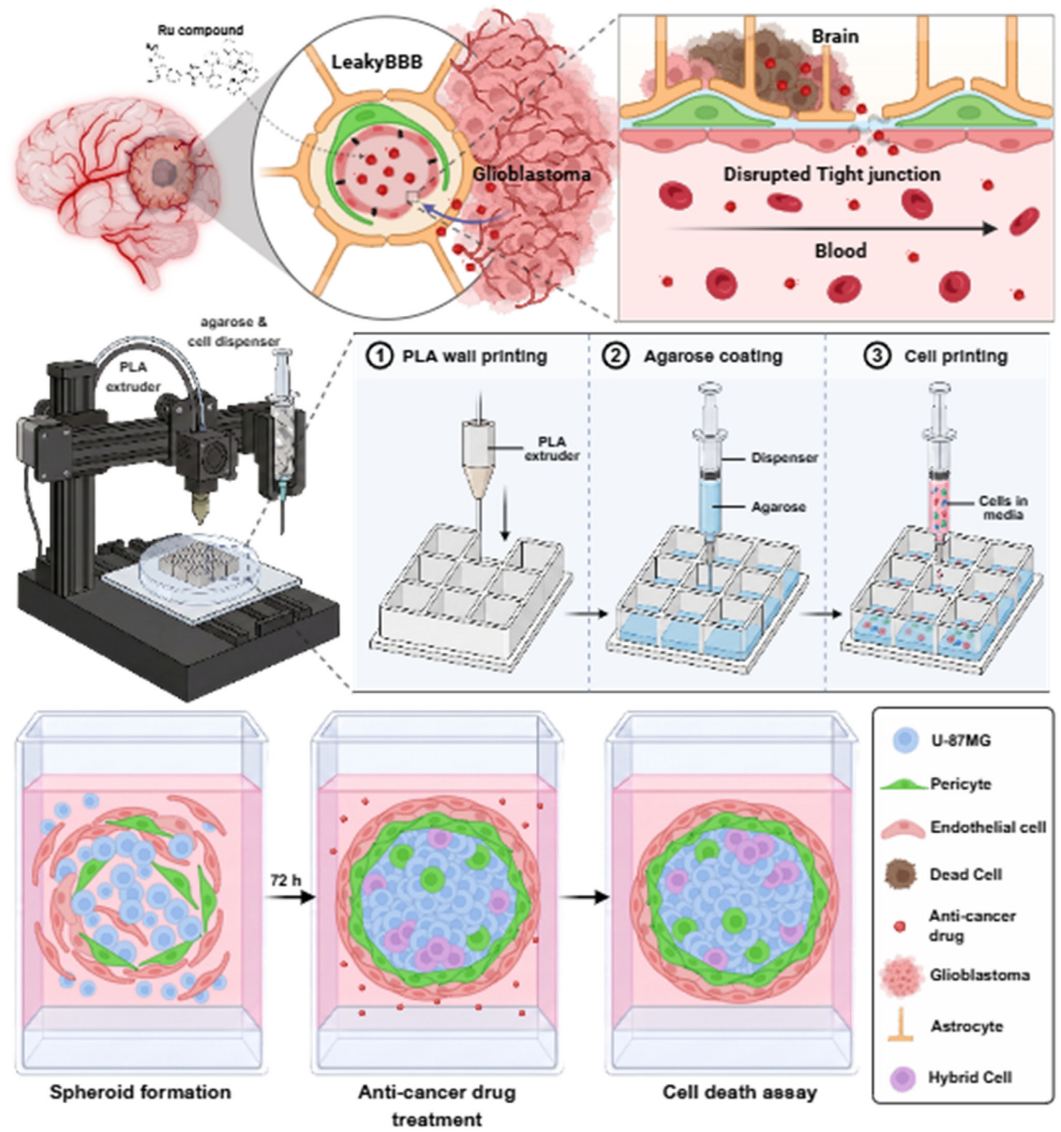
Overview of the BBTB microenvironment and the 3D bioprinting workflow for BBTB spheroid fabrication. (Top left) Schematic representation of the in vivo BBTB microenvironment in glioblastoma, illustrating the leaky BBB comprising brain endothelial cells, pericytes, astrocyte end‐feet, and GBM tumor cells, together with the chemical structure of the ruthenium (Ru) compound. (Top right) Enlarged view of the disrupted tight junction, illustrating passage of the Ru compound from blood into the brain parenchyma. (Middle) Bioprinting‐based fabrication workflow: PLA wall printing, agarose coating, and cell printing. (Bottom) Spheroid formation, anticancer drug treatment, and cell death assay. The BBTB spheroid is assembled from hCMEC/D3 endothelial cells, HBVPs, and U‐87 MG cells; astrocytes are depicted only in the in vivo schematic and are not a constituent of the fabricated BBTB spheroid. The legend indicates the cell types used. Created in BioRender. Song, J. M. (2026) https://BioRender.com/uv8dq98.

**FIGURE 2:**
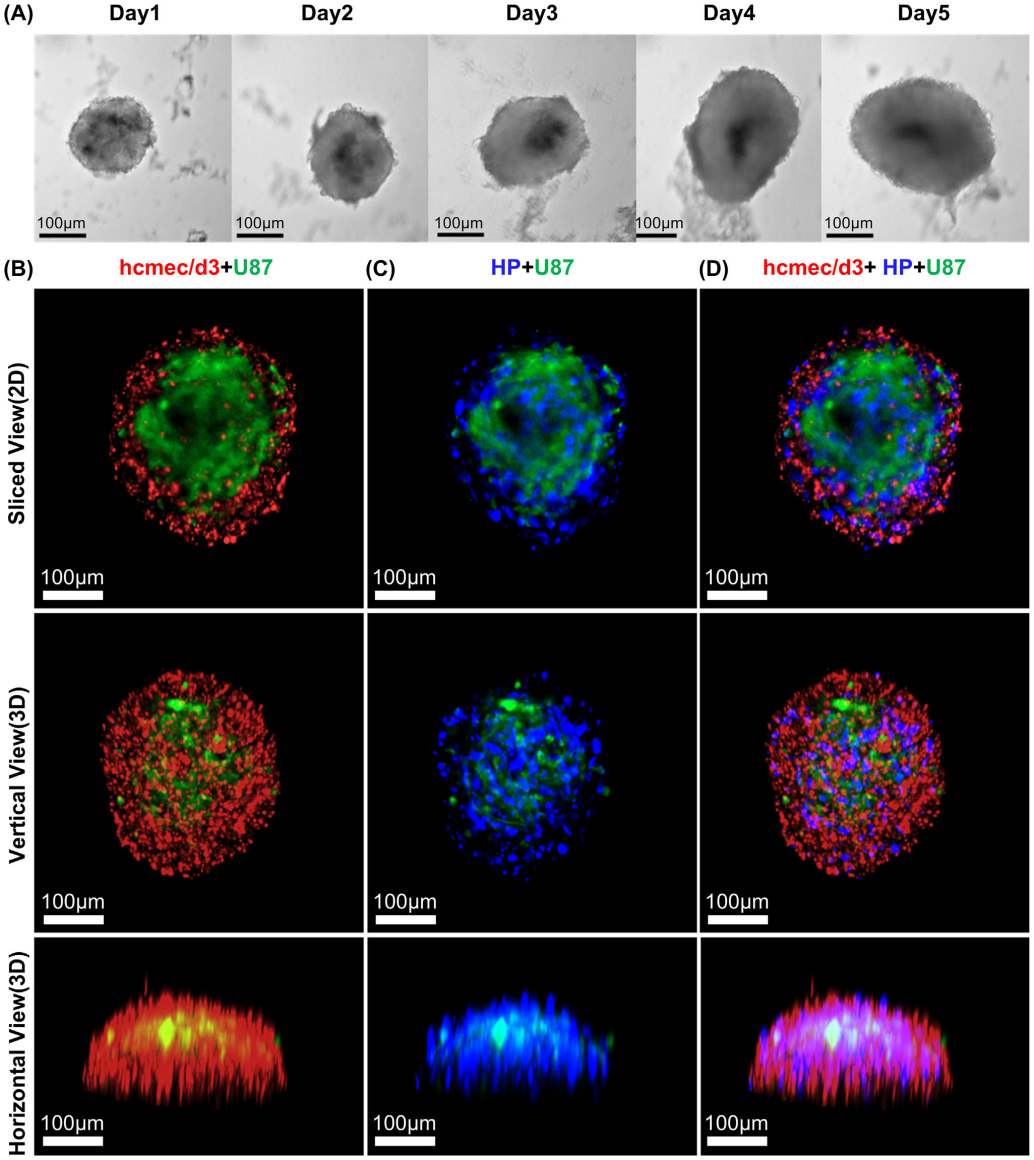
Morphogenesis and cellular organization of 3D bioprinted BBTB spheroids. (A) Bright‐field images of daily morphological changes in BBTB spheroids from Day 1 to Day 5. Scale bar = 100 µm. (B–D) Three‐dimensional renderings of confocal z‐stacks (sliced, vertical, and horizontal) of day‐3 spheroids composed of hCMEC/D3 and U‐87 MG (B), HBVPs and U‐87 MG (C), and all three cell types (D). Panels B–D show three different cell compositions, each represented by one spheroid; Spheroids from a single preparation (n = 3) were examined with consistent results. Scale bar = 100 µm.

Previous research indicates that cancer cells exhibit stronger adhesion to pericytes than to endothelial cells and that GBM utilizes cell–cell interactions with pericytes to facilitate tumor progression, or even cause cytoplasmic mixing [12, 13, 25]. We hypothesized that the invasion of pericytes into the U‐87 MG core resulted from cytoplasmic mixing resulting from the interaction between the two cells. To investigate this, HBVPs and U‐87 MG cells independently pre‐labeled with CellTracker dyes were co‐seeded on confocal dishes. At 12 h post‐seeding, their fluorescence signals remained spatially distinct, indicating no mixing (Figure 3A). However, at 48 h, both fluorescence signals were detected within individual cells, demonstrating cytoplasmic exchange between HBVPs and U‐87 MG cells (Figure 3B). BBTB spheroids were observed to determine whether this cytoplasmic mixing also occurs in spheroids. We examined cytoplasmic mixing across three independently fabricated spheroids, where only HBVPs and U‐87 MG cells were labeled. Cytoplasmic mixing was most prominent near the spheroid periphery, corresponding to the BBB‐like interface. Overlay images combining brightfield and fluorescence channels revealed regions of signal co‐localization, appearing as yellow or orange puncta, with color intensity reflecting the relative contributions of the red HBVP signal and green U‐87 MG signal (Figure 3C). These observations collectively confirm that cytoplasmic mixing between HBVPs and U‐87 MG cells occurs consistently and reproducibly within the BBTB spheroids.

**FIGURE 3:**
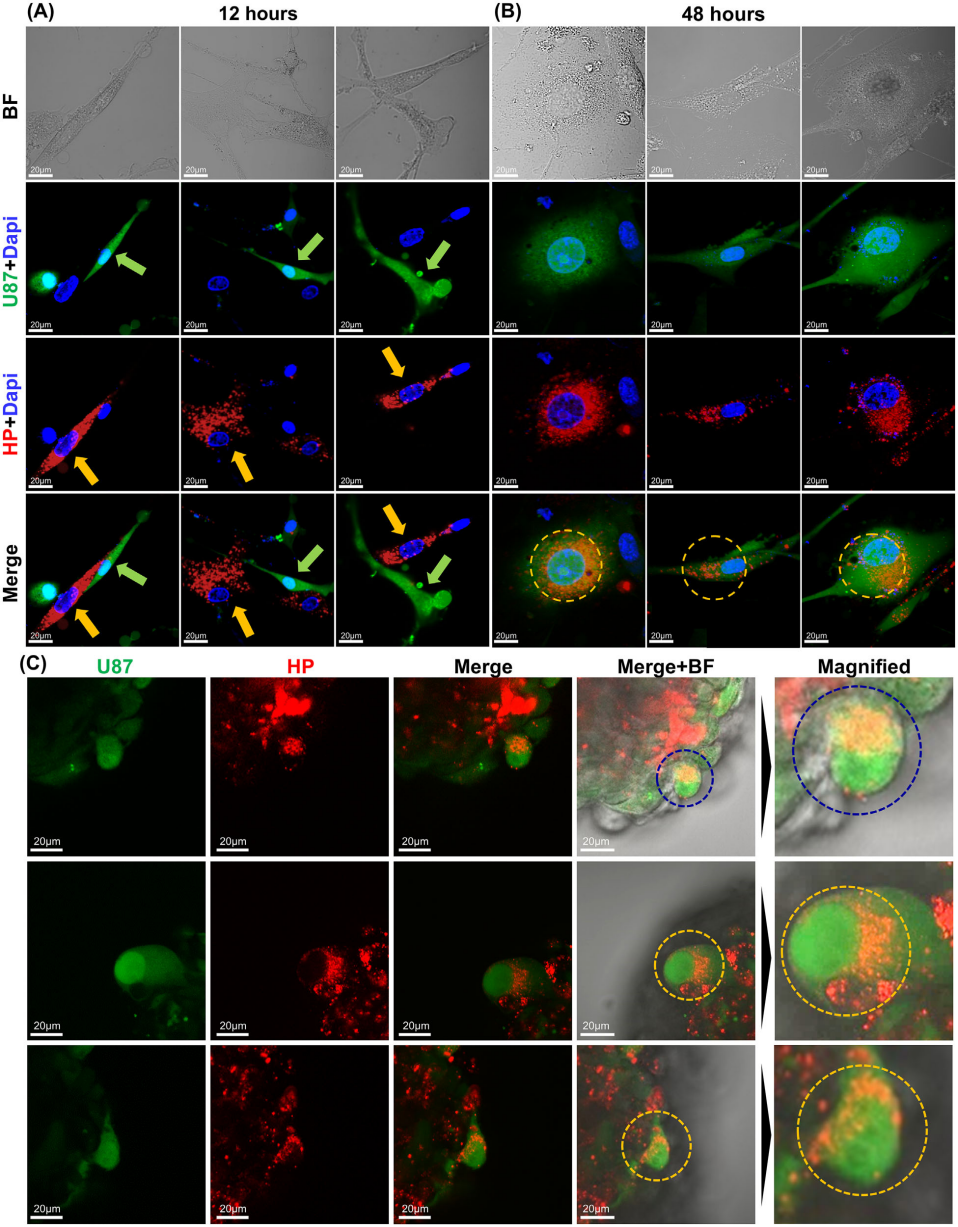
Cytoplasmic mixing of human pericyte (HP) and U‐87 MG cells. (A,B) Co‐culture of pre‐labeled human pericyte (HP; red) and U‐87 MG (green, DAPI‐stained nuclei in blue) cells at 12 h (A) and 48 h (B) after seeding on a confocal dish. Rows show bright‐field (BF), U‐87 MG + DAPI, HP + DAPI, and merged images. Arrows indicate regions of cytoplasmic contact. (C) Confocal images of BBTB spheroids showing U‐87 MG (green), HP (red), merge, merge + bright‐field (BF), and magnified views. The blue‐ and yellow‐dashed circles in the Merge + BF panels indicate regions enlarged in the Magnified column, demonstrating cytoplasmic mixing between HP and U‐87 MG cells. Scale bar = 20 µm. Each panel shows one representative image. For (A,B), three confocal dishes seeded in parallel were imaged separately; for (C), three separately formed spheroids from a single preparation were imaged separately. Consistent results were obtained in all cases.

The cells continue to proliferate even after day 3. To determine whether the layered arrangement is retained until day 5, spheroids prepared in the same way were cultured under the same conditions and imaged at day 5. The arrangement was preserved, with hCMEC/D3 cells forming the outermost layer, HBVPs occupying an intermediate position and U‐87 MG cells forming the core (Figure S1). Because CellTracker signal is diluted by cell division, these images indicate that the spatial organization is retained rather than that the population ratio is unchanged.

### 2.2 Expression of BBB‐Associated Proteins in Fabricated BBTB Spheroids

The BBB relies on TJ proteins—including ZO‐1, occludin, and claudin‐5—and the efflux transporter P‐gp to maintain barrier function [26]. TJ proteins create a physical seal between adjacent endothelial cells, limiting the movement of large molecules from the bloodstream into the brain parenchyma. In this study, expression of these BBB‐associated proteins in the BBTB spheroids was verified using immunofluorescence confocal microscopy (Figure 4A1–D1). Nuclei were counterstained with Hoechst 33342 (Figure 4A2–D2). Images were acquired at 8 µm intervals from the spheroid surface toward the core, reaching a depth of approximately 100–140 µm. This range covered the full depth over which the nuclear signal could be resolved. Overlaying the Hoechst 33342 staining with each TJ protein signal revealed the extent of co‐localization (Figure 4A3–D3); areas with intense Hoechst signals but weak protein signals indicated regions with lower TJ protein expression. The spatial distribution of TJ protein‐positive regions was rendered as a 3D surface (Figure 4A4–D4), with individual cells within TJ protein‐positive areas annotated as yellow dots, and cells outside these regions as red dots, to distinguish expressing from non‐expressing cells (Figure 4A5–D5). Figure 4E provides an enlarged view of Figure 4C5, illustrating the spatial distribution of yellow and red dots in greater detail. The number of TJ protein‐expressing cells was counted, and the percentage of total outer‐layer cells expressing them was statistically determined. Through this analysis, levels of TJ proteins, ZO‐1, occludin, and claudin, along with P‐gp, were evaluated (Figure 4F). All four BBB‐related proteins were robustly expressed in the BBTB spheroids. To identify the cell type exhibiting these signals, spheroids were assembled with CellTracker‐labeled hCMEC/D3 cells and immunostained for each protein. The protein signals colocalized with the labeled endothelial cells (Figure S2).

**FIGURE 4:**
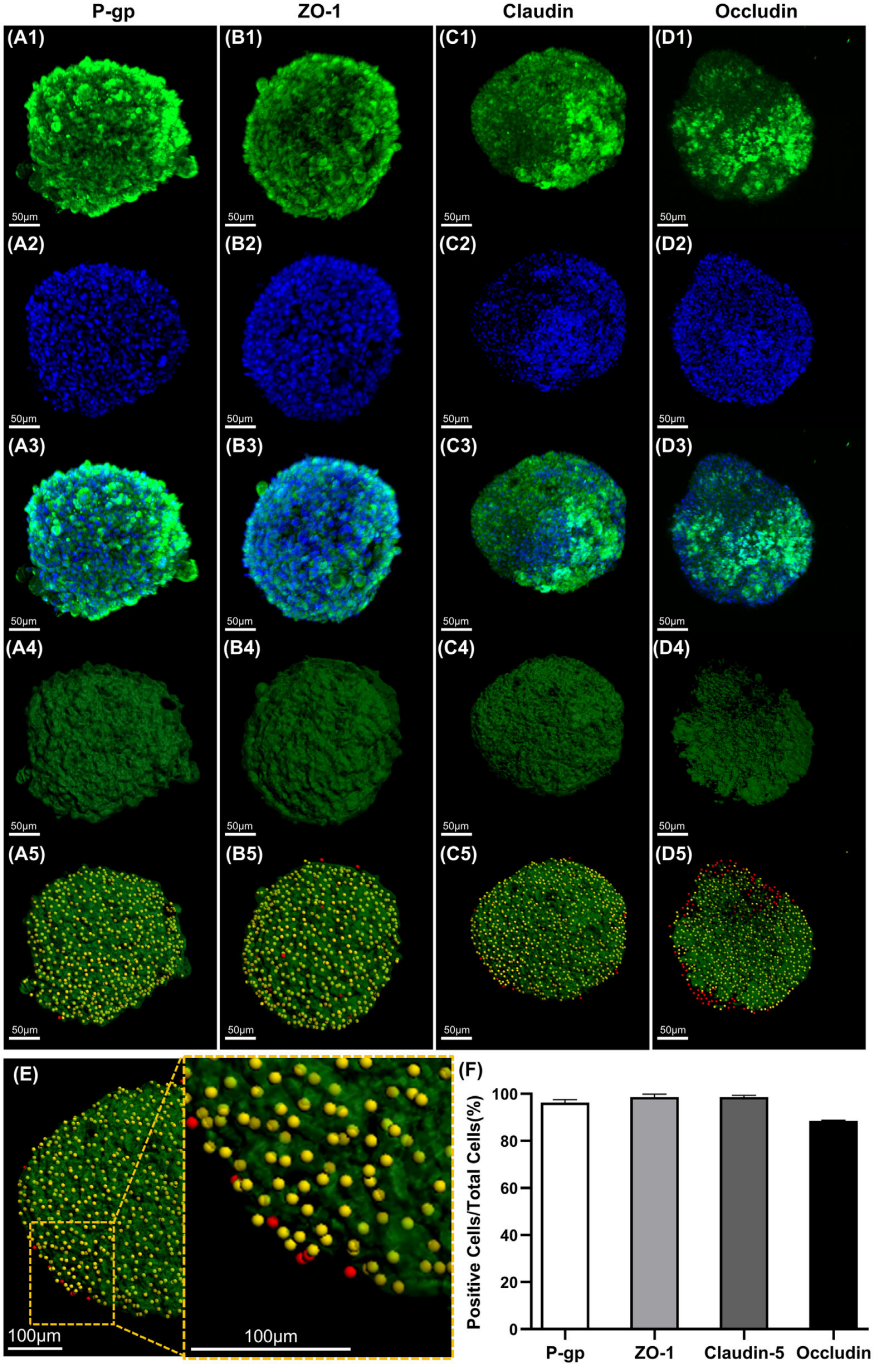
Expression and distribution of BBB‐associated proteins in BBTB spheroids. (A1–D5) Confocal immunofluorescence analysis of BBB‐associated proteins in BBTB spheroids: P‐glycoprotein (A), ZO‐1 (B), Claudin‐5 (C), and Occludin (D). For each protein, the following images are shown: immunofluorescence signal (1), Hoechst 33342 nuclear staining (2), merged image (3), three‐dimensional rendering of the protein‐positive surface area (4), and spatial mapping of protein‐expressing cells (yellow dots) versus non‐expressing cells (red dots) calculated based on the distance between the nucleus and the protein‐positive area (5). (E) Enlarged view of panel C5, showing the cell‐level distribution of claudin‐5 in greater detail. (F) Percentage of outer‐layer cells expressing each protein. Data in (F) are presented as mean ± SD of n = 2 independent experiments, each comprising two separately formed spheroids that were averaged before analysis (four spheroids in total). No statistical comparison was performed between proteins. Scale bar = 50 µm in panels A1‐D5 and 100 µm in panel E.

### 2.3 Permeability Comparison Among U‐87 MG, BBB, and BBTB Spheroids Using Dextran‐FITC

The BBB is formed by TJ complexes between brain endothelial cells, serving as a physical blockade that determines which molecules can move between the bloodstream and the brain, particularly hydrophilic macromolecules. In GBM cases, the BBTB develops and becomes more permeable than the typical BBB due to tumor‐induced VEGF upregulation, fewer pericytes, reduced TJ proteins, and impaired connections between astrocyte end‐feet and the endothelial layer [26, 27, 28]. To determine whether the fabricated spheroid model recapitulates these differences in permeability, FITC‐conjugated dextran (Dextran–FITC, 40 kDa)—a representative hydrophilic macromolecular tracer—was applied to three spheroid types: U‐87 MG‐only, BBB, and BBTB. Using confocal microscopy, cross‐sectional images of Dextran–FITC fluorescence were acquired at 8 µm intervals from the spheroid surface to a depth of 96 µm along the z‐axis. The fluorescence distribution of Dextran–FITC in U‐87 MG (Figure 5A1–A8), BBTB (Figure 5B1–B8), and BBB (Figure 5C1–C8) spheroids was analyzed across depths of 8–96 µm (Figure 5D). As described in Section 2.1, the BBB spheroids contain astrocytes in place of the tumor cells present in the BBTB spheroids; the two constructs therefore differ both in the presence of tumor cells and in the presence of astrocytes.

**FIGURE 5:**
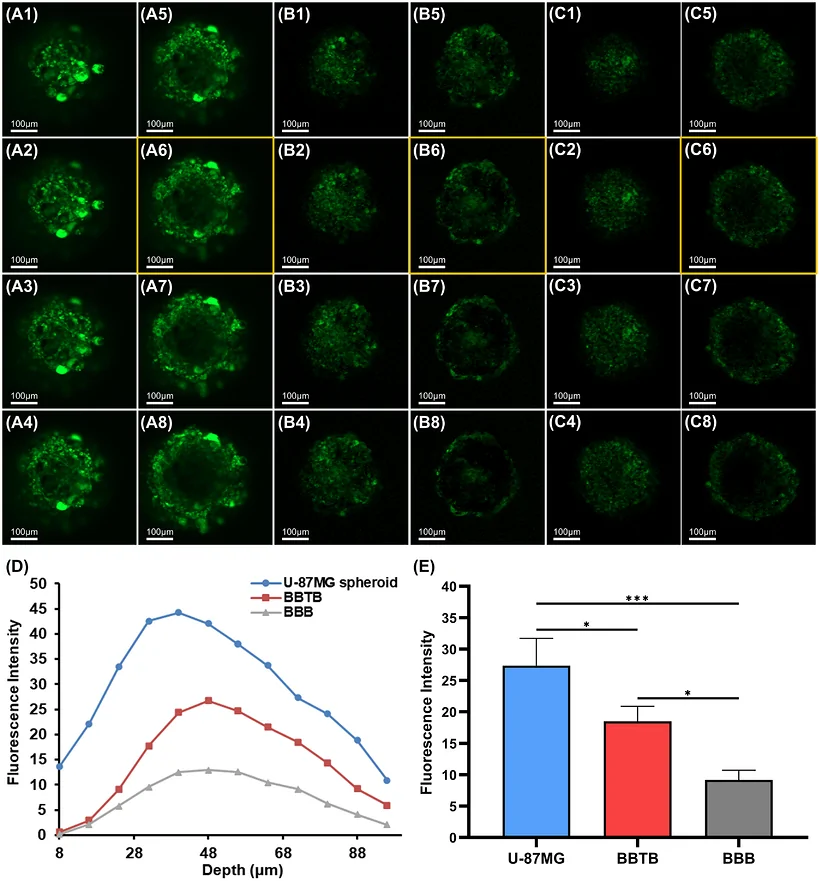
Permeability comparison of dextran‐FITC across U‐87 MG, BBTB, and BBB spheroids. (A1–A8, B1–B8, C1–C8) Serial cross‐sectional confocal images of dextran‐FITC fluorescence acquired at multiple depths from U‐87 MG spheroids (A), BBTB spheroids (B), and BBB spheroids (C). (D) Depth‐resolved dextran‐FITC fluorescence intensity profiles for each spheroid type, with fluorescence intensity (y‐axis) plotted against imaging depth (x‐axis, 8–96 µm). (E) Dextran‐FITC fluorescence intensity among the three spheroid types at 72 µm depth. Scale bar = 100 µm. Data are presented as mean ± SD of n = 3 separately formed and treated spheroids per group from a single independent experiment. Statistical significance was assessed by ordinary one‐way ANOVA with Tukey's multiple‐comparisons test (two‐sided, alpha = 0.05). *p < 0.05, ***p < 0.001. GraphPad Prism 11.0.2.

At identical imaging depths, U‐87 MG spheroids—lacking an endothelial barrier—demonstrated the highest Dextran–FITC fluorescence, confirming greater macromolecular penetration in the absence of a TJ‐based barrier. Fluorescence intensities at the representative depth of 72 µm were compared using a bar graph (Figure 5E). As expected, BBTB spheroids showed lower permeability to Dextran–FITC than U‐87 MG spheroids, but slightly higher permeability than BBB spheroids (Figure 5C–E). This trend was consistent across all tested depths. Collectively, these results suggest that the fabricated BBTB spheroids possess a physical barrier to hydrophilic macromolecules and exhibit the characteristic “leaky” properties associated with BBTB in vivo.

### 2.4 Anticancer Effect of Ru Compound

Understanding the subcellular localization of anticancer agents within cancer cells is crucial for elucidating the anticancer mechanism of ROS‐generating compounds. As ROS have lifespans measured in nanoseconds, the organelles where these species are generated cannot avoid their destructive effects. To determine the intracellular distribution of the Ru compound, subcellular localization assays were conducted using U‐87 MG cells. MitoTracker Red and ER‐Tracker Red identified whether the Ru compound accumulated within the mitochondria and ER, respectively. Confocal microscopy analysis indicated that the Ru compound was present in the mitochondria and ER (Figure 6A,B), suggesting that its mechanism of action involves these organelles. Single‐label controls indicating that neither fluorophore contributes appreciably to the channel assigned to the other are provided in Figure S3. Transition‐metal complexes, including Ru compounds, generate ROS via Fenton‐type reactions [29]. Their redox cycling can deactivate antioxidants while promoting ROS formation in the acidic environment's characteristic of cancer cells. Due to enhanced metabolism, cancer cells generate more ROS than normal cells, primarily due to rapid metabolism, which promotes oxidative phosphorylation [30, 31]. To mitigate elevated ROS levels, these cells upregulate antioxidants such as glutathione (GSH) and thioredoxin [32, 33]. Hence, transition‐metal complex‐based drugs that generate ROS can disturb this balance, leading to oxidative damage and apoptosis. Additionally, Ru complexes induce apoptosis by increasing intracellular ROS in cancer cells [34].

**FIGURE 6:**
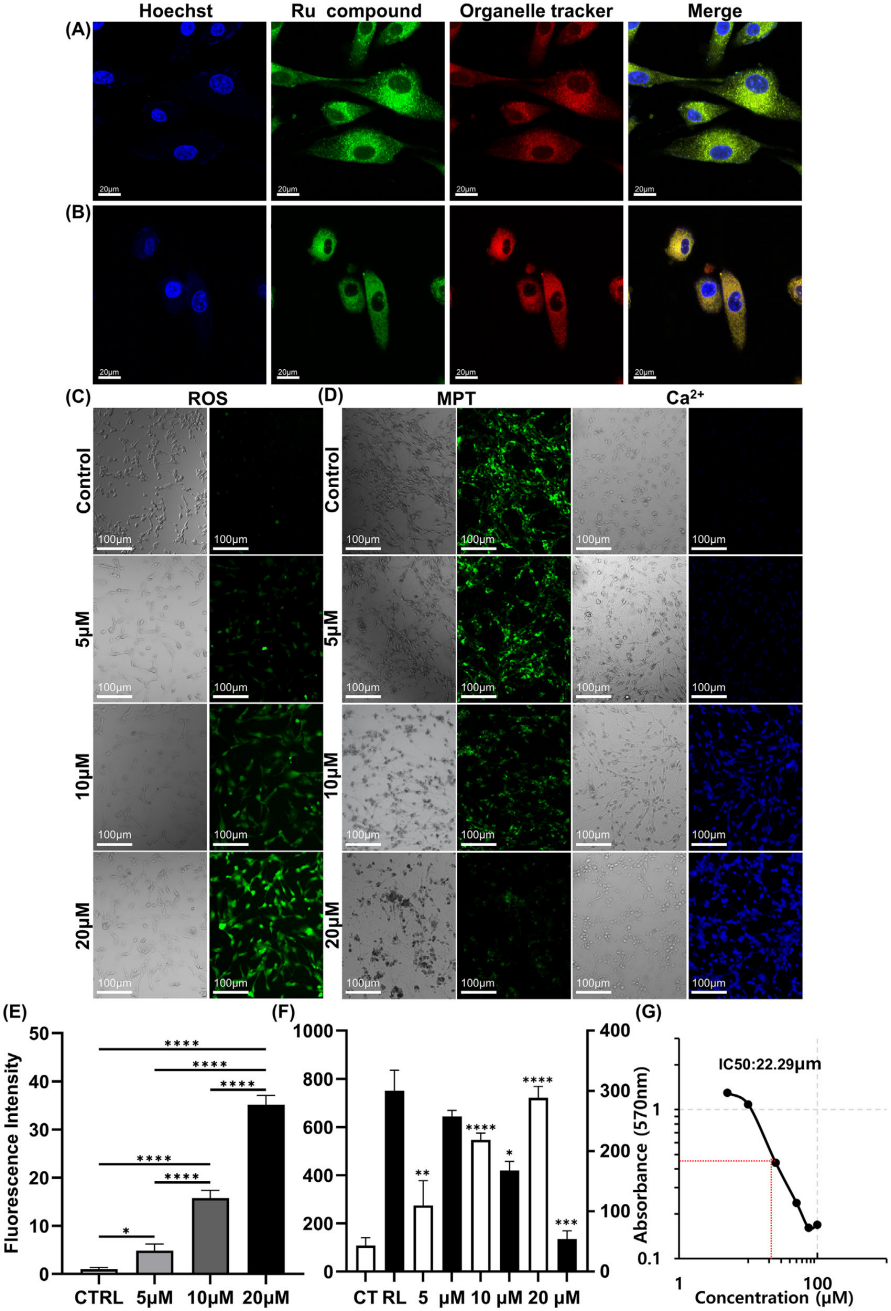
Subcellular localization of the Ru compound and its effects on U‐87 MG cells. (A,B) Confocal images of mitochondrial (A) and endoplasmic reticulum (ER) (B) localization of the Ru compound (green) co‐stained with organelle‐specific trackers (red) and Hoechst 33342 nuclear stain (blue) in U‐87 MG cells. (C) Intracellular ROS generation assessed by DCFDA fluorescence (green) in U‐87 MG cells treated with the Ru compound at 0, 5, 10, and 20 µM. (D) Mitochondrial permeability transition (MPT) assessed by Calcein AM (green) and intracellular calcium (Ca2+) assessed by Indo‐1 (blue) in U‐87 MG cells at the same concentrations. (E) Quantified DCFDA fluorescence intensity across treatment groups. (F) Calcein AM (MPT) and Indo‐1 (Ca2+) fluorescence intensity. (G) Dose–response curve from MTT assay showing the cytotoxic effect of the Ru compound on U‐87 MG cells with an IC_50_ of 22.3 µM. Scale bar = 20 µm in panels A and B; 100 µm in panels C and D. Images in (A–D) are representative of three confocal dishes seeded in parallel per condition. Data in (E,F) are the quantification of the three dishes shown in (C) and (D) and are presented as mean ± SD of n = 3 dishes seeded in parallel, with one measurement per dish; data in (G) are presented as mean ± SD of n = 3 replicate wells per concentration seeded in parallel. Statistical significance in (E, F) was assessed by ordinary one‐way ANOVA with Tukey's multiple‐comparisons test (two‐sided, alpha = 0.05); *p < 0.05, **p < 0.01, ***p < 0.001, ****p < 0.0001; n.s., not significant. The IC_50_ in (G) was obtained by non‐linear regression. GraphPad Prism 11.0.2.

DCFDA is a ROS‐sensing material that emits green fluorescence on reacting with ROS. We used DCFDA to verify whether the Ru compound generates ROS in U‐87MG cells. The fluorescence intensity of DCFDA, reflecting ROS levels, increased with Ru treatment when its concentration was increased from 5 to 20 µM (Figure 6C). The fluorescence intensity of DCFDA was plotted as a function of treated concentration of Ru compound in Figure 6E. These results demonstrate that the Ru compound possesses anticancer activity, acting notably through mitochondrial and the ER damage via ROS induction. Considering ROS generation ability of the Ru compound and its localization in the ER and mitochondria, additional studies were performed to assess MPT and disruption of Ca^2+^ homeostasis—both associated with cancer cell death induced by Ru compounds. The ER serves as a reservoir for intracellular calcium and provides an environment conducive to proper protein folding via accessory proteins such as GRP‐78 chaperone [35, 36]. Excessive ROS induces oxidative stress, prompting Ca^2+^ release from the ER into the cytoplasm, thereby disrupting Ca^2+^ homeostasis and ultimately leading to cell death. MPT, characterized by increased inner mitochondrial membrane permeability, occurs when excessive increases in ROS and intracellular Ca^2+^ trigger opening of the mitochondrial permeability transition pore (mPTP). This causes water influx into the mitochondrial matrix, resulting in organelle collapse [37, 38]. The release of cytochrome c subsequently activates caspases, ultimately inducing apoptosis [39]. We evaluated the Ru compound‐induced cytotoxic mechanism (Figure 6D). Intracellular MPT was measured using Calcein AM and CoCl_2_. Calcein AM passively diffuses into cells and accumulates in intracellular compartments, including mitochondria. Within the cell, intracellular esterase cleaves the AM ester, liberating calcein, a very polar fluorescent dye, which can no longer penetrate cell membranes or mitochondria. CoCl_2_ quenches cytosolic Calcein AM fluorescence, isolating the mitochondrial signal. Therefore, Calcein AM fluorescence is inversely proportional to the intracellular MPT. Results revealed diminished intracellular calcein fluorescence and increased cytosolic Ca^2+^ proportional to the Ru compound concentration (Figure 6D). Corresponding emission intensities for MPT and cytosolic Ca^2+^ were shown as a function of Ru concentration (Figure 6F). These outcomes confirm that the Ru compound induces cytosolic Ca^2+^ imbalance and MPT in U‐87MG cells. Finally, cytotoxicity of the Ru compound was assessed via MTT assays (Figure 6G), revealing a 50% reduction in U‐87 MG cell viability at a Ru concentration of 22.3 µM.

### 2.5 Effects of the Ru Compound on BBB‐Related Proteins

Redox regulation is essential for cytoskeleton proteins with redox‐sensitive sites, which play a critical role in pericytes that comprise the BBB. These pericytes express proteins such as α‐SMA, myosin, and tropomyosin, which are upregulated as ROS concentrations increase. Alterations in cytoskeletal proteins can prompt pericyte constriction, thereby affecting BBB permeability [40]. MMP‐9 is a zinc‐dependent protease that degrades components of the ECM and basement membrane. MMP expression has also been associated with superoxide dismutase (SOD) activity, which mitigates oxidative stress. Elevated ROS levels induce increased MMP expression in cells such as astrocytes and endothelial cells; excessive MMPs impact not only the ECM but also TJ proteins such as occludin and ZO‐1, leading to increased BBB permeability [20, 40, 41, 42]. Given the effect of ROS on the BBB and the Ru compound's propensity to generate ROS, the effects of the Ru compound on BBB integrity were investigated. Cytoskeletal alterations in pericytes, along with biomarkers of BBB disruption, such as MMP‐9 and ET‐1, were observed using immunostaining and ELISA. Figure 7A showed the change in cytoskeleton of pericytes caused by the Ru compound. Compared with the control, levels of a‐SMA and F‐actin were increased in pericytes following treatment with the Ru compound. This upregulation may promote contractility, enhance migratory and invasive properties, further vasoconstriction, and compromise BBB integrity [20, 40, 43]. Figure 7B showed the quantitative comparison between control and Ru treatment on the expression of a‐smooth muscle actin and F‐actin in pericytes. Immunofluorescence analysis demonstrated increased MMP‐9 expression in both hCMEC/D3 endothelial cells (left panel, Figure 7C) and astrocytes (right panel, Figure 7C) following Ru compound treatment compared with the vehicle controls. Quantitative comparisons of MMP‐9 fluorescence intensity are shown in Figure 7D (hCMEC/D3 cells) and Figure 7E (astrocytes). ELISA data further corroborated a concentration‐dependent increase of secreted MMP‐9 in hCMEC/D3 cells (Figure 7F) and astrocytes (Figure 7G). ET‐1, a potent vasoconstrictor produced by vascular endothelial cells, increases BBB permeability and impairs its integrity. Accordingly, ET‐1 serves as a biomarker for BBB disruption and is predominantly expressed in activated astrocytes [44]. It induces ROS production through activation of NOX and COX, promoting vasoconstriction, increasing BBB permeability, and decreasing p‐gp function. ET‐1 expression increases in response to oxidative stress [42, 45]. The Ru compound augments intracellular oxidative stress by generating ROS. ELISA results demonstrated a significant, concentration‐dependent increase in ET‐1 secretion by astrocytes in the presence of 5 µM to 20 µM Ru, compared with vehicle controls (Figure 7H).

**FIGURE 7:**
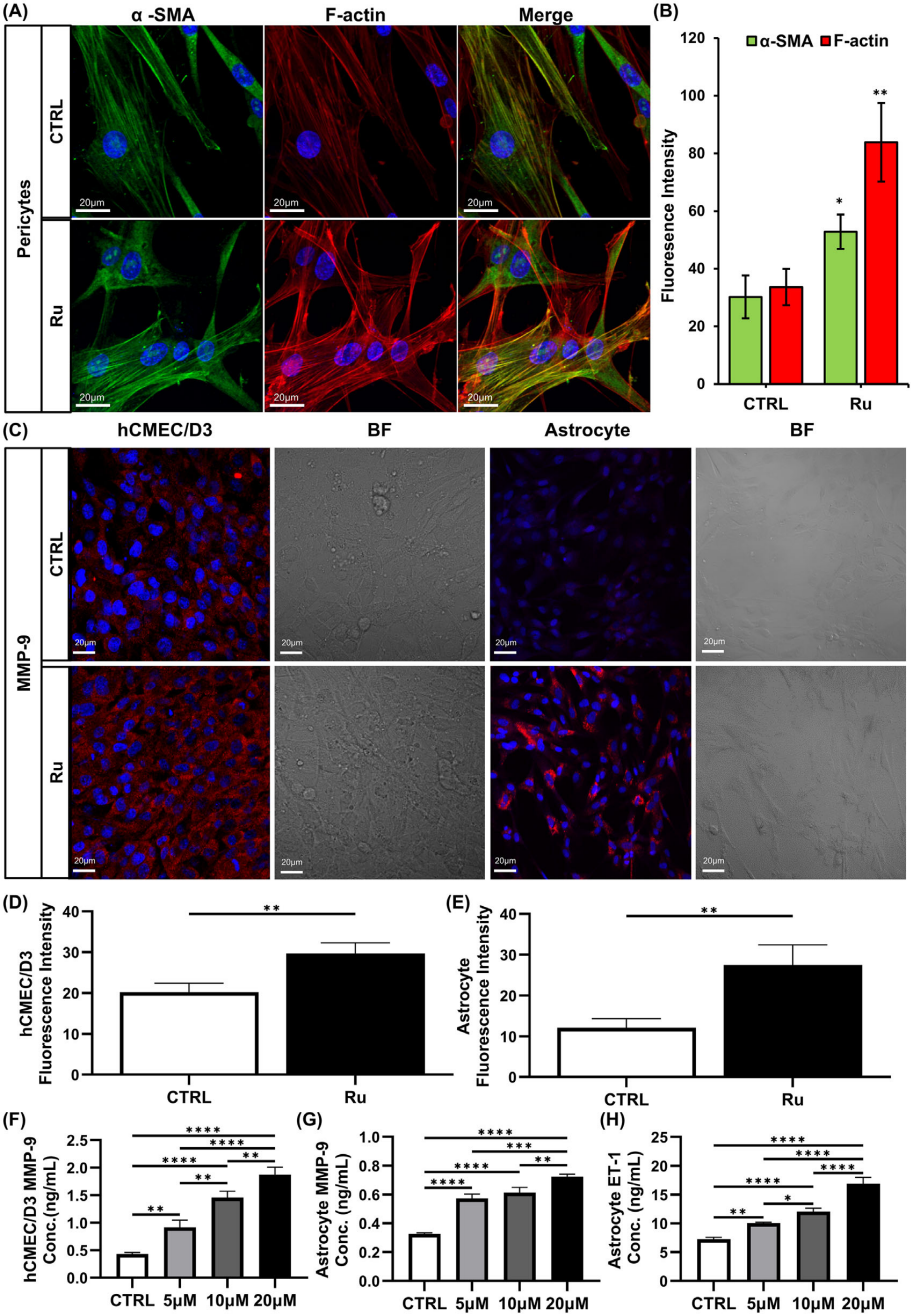
Effects of the Ru compound on BBB‐associated protein expression. (A) Immunofluorescence images of α‐SMA (green) and F‐actin (red) in pericytes treated with vehicle control (top) or the Ru compound (10 µM, bottom). Nuclei are stained with Hoechst 33342. (B) Fluorescence intensity changes of α‐SMA and F‐actin expression in control and Ru‐treated pericytes. (C) Immunofluorescence images of MMP‐9 (red) and bright‐field (BF) in hCMEC/D3 cells (left two columns) and astrocytes (right two columns) treated with vehicle control or Ru compound (20 µM). Nuclei are stained with Hoechst 33342 (blue). (D–E) MMP‐9 fluorescence intensity in hCMEC/D3 cells (D) and astrocytes (E). (F–H) ELISA quantification of secreted MMP‐9 in hCMEC/D3 cells (F) and astrocytes (G), and endothelin‐1 in astrocytes (H), following Ru compound treatment at 5, 10, and 20 µM. Scale bars = 20 µm. Images in (A) and (C) are representative of three confocal dishes seeded in parallel per condition, and the quantification in (B), (D) and (E) is derived from those three dishes (n = 3). Data in (F–H) are from n = 3 wells seeded and treated in parallel, with a single ELISA measurement per well. Data are presented as mean ± SD. Statistical significance in (B), (D) and (E) was assessed by an unpaired two‐tailed Student's t‐test, and in (F–H) by ordinary one‐way ANOVA with Tukey's multiple‐comparisons test; all tests used a two‐sided alpha of 0.05. *p < 0.05, **p < 0.01, ***p < 0.001, ****p < 0.0001. Analyses were performed using GraphPad Prism 11.0.2.

### 2.6 Ru compound Compromises TJ Protein Expression

TJs are multi‐protein complexes that serve as the primary paracellular barrier between adjacent brain endothelial cells. The proteins comprising TJs are predominantly divided into two main categories. First, the integral membrane proteins (including claudin and occludin families) bridge the gaps between cells. Second, peripheral proteins (zonula occludens (ZO) family) on the cytoplasmic side of the synaptic region interact directly or indirectly with integral membrane proteins [44, 46, 47]. TJs prevent macromolecules from passing between cells, forcing these molecules to enter endothelial cells via endocytosis to penetrate the brain. Hence, the BBB restricts the free diffusion of macromolecules between the blood and brain, permitting only ions and small hydrophobic molecules (< 500 Da) to pass through [48].

To examine how Ru‐induced ROS affect TJ proteins, changes in intracellular TJ protein expression following Ru compound treatment were investigated. Immunofluorescence images revealed a marked decrease in occludin and ZO‐1 protein levels when endothelial cells were treated with 20 µM Ru compound (Figure 8A). The immunofluorescence intensities of occludin and ZO‐1 protein were plotted in a bar graph to quantitatively show the decreased expression level compared with the control (Figure 8B). Western blot analysis further confirmed concentration‐dependent suppression of ZO‐1 and occludin at 10 and 20 µM Ru compound, with statistically significant downregulation at 20 µM for both proteins (Figure 8C,D). These results suggest that the Ru compound might compromise BBB integrity by affecting TJ proteins.

**FIGURE 8:**
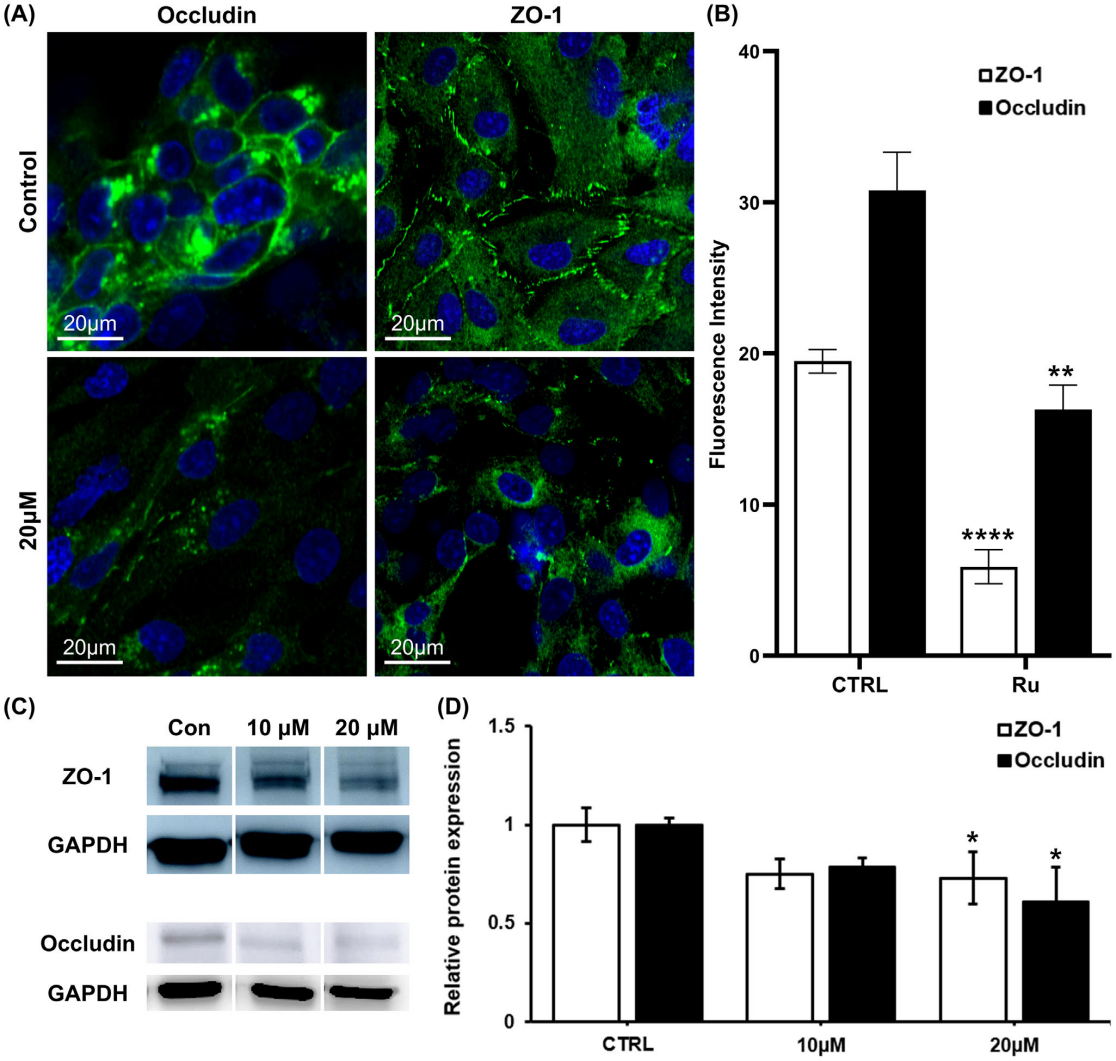
Effects of the Ru compound on tight junction protein expression in hCMEC/D3 endothelial cells. (A) Immunofluorescence images of occludin and ZO‐1 in hCMEC/D3 cells treated with vehicle control (top) and the Ru compound (20 µM, bottom). Nuclei are stained with Hoechst 33342 (blue). (B) Fluorescence intensity of occludin and ZO‐1 under control and Ru compound‐treated conditions. (C) Representative western blot images showing ZO‐1 and occludin protein expression in hCMEC/D3 cells treated with vehicle control, 10, or 20 µM Ru compound. GAPDH was used as a loading control. (D) Relative protein expression of ZO‐1 and occludin normalized to GAPDH. Scale bar = 20 µm. Uncropped blot images are provided in Figure S4. Images in (A) are representative of three confocal dishes seeded in parallel per condition, and the quantification in (B) is derived from those three dishes (n = 3). For (C,D), lysates were prepared from three flasks seeded on separate days and resolved as three lanes per condition on a single membrane (n = 3 independent biological replicates). Data are presented as mean ± SD. Statistical significance was assessed separately for each protein: in (B) by an unpaired two‐tailed Student's t‐test, and in (D) by ordinary one‐way ANOVA with Tukey's multiple‐comparisons test; all tests used a two‐sided alpha of 0.05.*p < 0.05, **p < 0.01, ***p < 0.001, ****p < 0.0001; n.s., not significant. Analyses were performed using GraphPad Prism 11.0.2.

### 2.7 ROS Dependence of Ru‐Induced Changes in Barrier‐Associated Markers

The changes in tight junction proteins, MMP‐9 and ET‐1 described above were observed following exposure to a ROS‐generating compound. To determine whether these changes depend on ROS generation, hCMEC/D3 cells and primary human astrocytes were pretreated with the ROS scavenger N‐acetylcysteine (NAC, 5 mM, 2 h), after which the NAC‐containing medium was removed and the cells were washed before exposure to the Ru compound (20 µM, 48 h). Four groups were compared: vehicle‐treated control, NAC alone, Ru alone, and NAC pretreatment followed by Ru.

Ru exposure increased MMP‐9 secretion 3.0‐fold in both hCMEC/D3 cells (2.64 ± 0.42 versus 0.88 ± 0.24 ng mL^−^^1^) and astrocytes (0.36 ± 0.02 versus 0.12 ± 0.04 ng mL^−^^1^), and NAC pretreatment reduced these levels to 1.43 ± 0.27 and 0.19 ± 0.01 ng mL^−^^1^, respectively (Figure 9A,B). ET‐1 secretion by astrocytes was increased 2.9‐fold by Ru (16.89 ± 1.07 versus 5.85 ± 0.61 ng mL^−^^1^) and reduced to 8.40 ± 2.53 ng mL^−^^1^ by NAC pretreatment (Figure 9C). In each case, NAC pretreatment abolished 69%–77% of the Ru‐induced increase, whereas NAC alone did not alter basal secretion of these mediators.

**FIGURE 9:**
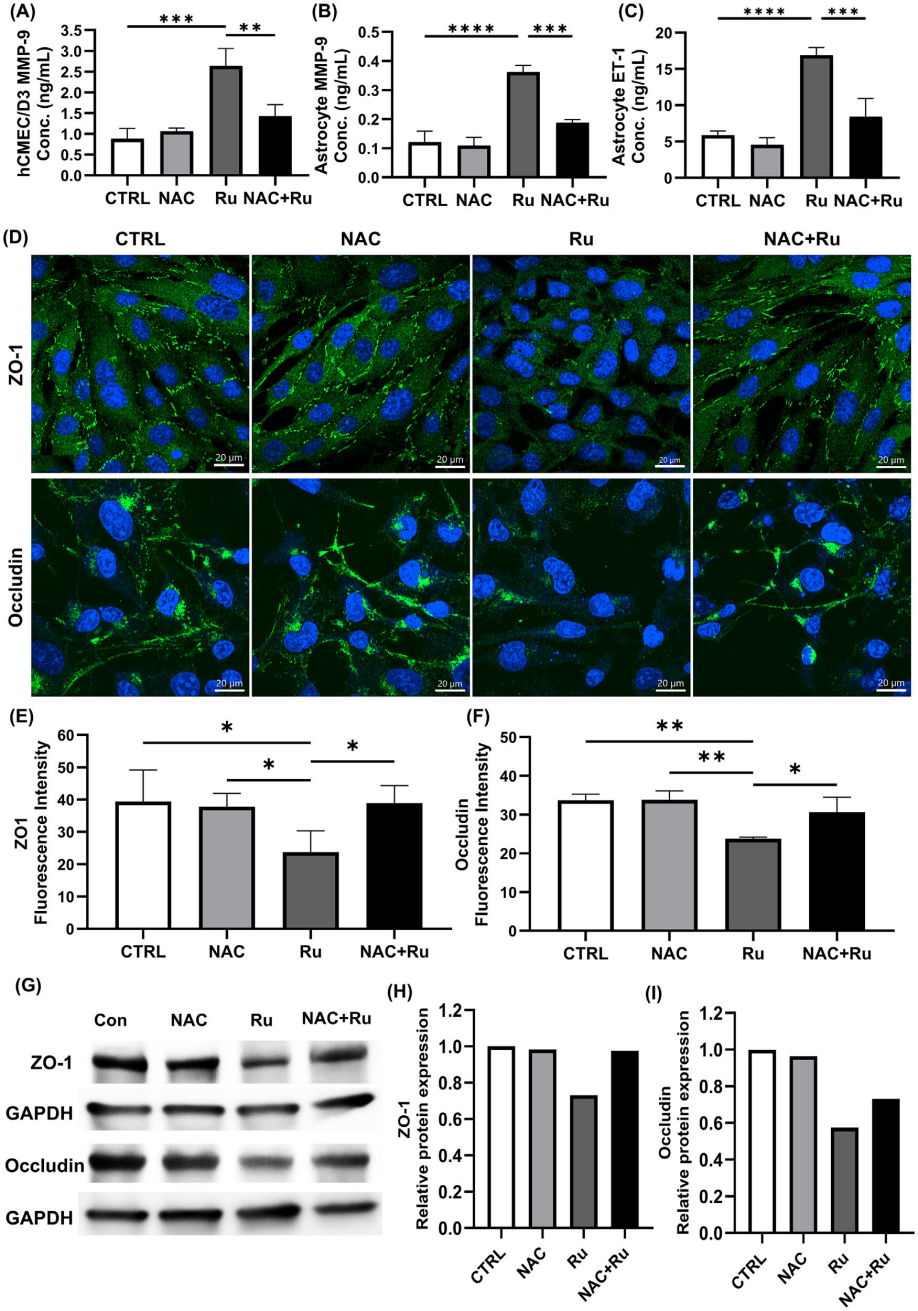
ROS dependence of Ru‐induced changes in barrier‐associated markers in hCMEC/D3 cells and astrocytes. Cells were pretreated with N‐acetylcysteine (NAC, 5 mM, 2 h), after which the NAC‐containing medium was removed and the cells were washed before exposure to the Ru compound (20 µM, 48 h). (A) MMP‐9 secretion by hCMEC/D3 cells, (B) MMP‐9 secretion by astrocytes, and (C) ET‐1 secretion by astrocytes, determined by ELISA. (D) Immunofluorescence images of ZO‐1 (top) and occludin (bottom) in hCMEC/D3 cells. Nuclei are stained with Hoechst 33342 (blue). Scale bars = 20 µm. (E,F) Quantitative analysis of ZO‐1 (E) and occludin (F) fluorescence intensity. (G) Representative immunoblot images of ZO‐1 and occludin, with GAPDH detected on the same membrane as a loading control following stripping; uncropped images are provided in Figure S4. (H,I) Relative protein expression of ZO‐1 (H) and occludin (I), normalized to the GAPDH signal in the corresponding lane and expressed relative to the vehicle‐treated control. Data in (A–C) are from n = 3 wells seeded and treated in parallel, with a single ELISA measurement per well. Images in (D) are representative of three confocal dishes seeded in parallel per condition, and the quantification in (E,F) is derived from those three dishes (n = 3). Data in (A–C,E,F) are presented as mean ± SD, and each panel was analyzed separately by ordinary one‐way ANOVA with Tukey's multiple‐comparisons test (two‐sided, alpha = 0.05); *p < 0.05, **p < 0.01, ***p < 0.001, ****p < 0.0001. The immunoblot in (G–I) was obtained from a single experiment with one lane per condition, each prepared from a separate lysate; the values in (H,I) are therefore shown as an independent confirmation of (E,F), and no statistical comparison was performed. Analyses were performed using GraphPad Prism 11.0.2.

NAC pretreatment also preserved tight junction protein expression in hCMEC/D3 cells. ZO‐1 immunofluorescence intensity, which decreased to 60.2% of the vehicle‐treated control after Ru exposure, was restored to 98.9% of control, and occludin intensity was restored from 70.5% to 90.6% of control; both recoveries were statistically significant (Figure 9D–F). Immunoblot analysis showed a corresponding pattern (Figure 9G–I). NAC alone did not alter basal levels of any of these markers. Together, these findings indicate that the changes in tight junction proteins, MMP‐9 and ET‐1 accompanying Ru exposure are redox‐dependent and can be prevented by antioxidant pretreatment.

### 2.8 Comparative Penetration of the Ru Compound Across U‐87 MG, BBB, and BBTB Spheroids

Given that the Ru compound induces ROS production and affects BBB‐related markers and TJ proteins, its impact on BBB integrity was investigated. Specifically, a permeability assay was conducted using the U‐87 MG‐only, BBB, and BBTB spheroids, and the distribution of the Ru compound was compared with that of the hydrophilic macromolecular tracer dextran. Fluorescence intensities of the Ru compound were measured in 2D cross sections of each spheroid where the Ru compound had penetrated, following the same standards as the dextran photographs. Based on the distributions of the Ru compound in the U‐87 MG, BBTB, and BBB spheroids at depths of 32–88 µm (Figure 10A–C) and 8–96 µm (Figure 10D) from the surface, the Ru compound successfully passed through all three spheroids. This was further inferred from the markedly brighter fluorescence intensities of the Ru compound compared with dextran (Figure 5A–C). Additionally, the fluorescence intensities of the penetrated Ru compound were similar across the three spheroids at a depth of 72 µm (Figure 10E). The overall sum and average fluorescence intensities of Ru and dextran at each depth were calculated across the three spheroids. Distribution differences between the Ru compound and dextran in the three spheroids were assessed by comparing the ratio of average fluorescence values for each spheroid type (Figure 10F). For dextran, the average fluorescence in U‐87 MG spheroids was approximately 3.5 times higher than in BBB spheroids. In contrast, the Ru compound yielded a ratio of 1.13 between U‐87 MG and BBB spheroids. Similarly, the average fluorescence ratios of dextran and the Ru compound between the BBTB and BBB spheroids were 2.05 and 1.05, respectively. Comparisons between U‐87 MG and BBTB spheroids showed ratios of 1.76 for dextran and 1.08 for the Ru compound, both demonstrating a consistent reduction trend. These findings suggest that the Ru compound, which generates ROS, alters BBB integrity and enhances permeability in BBB and BBTB spheroids.

**FIGURE 10:**
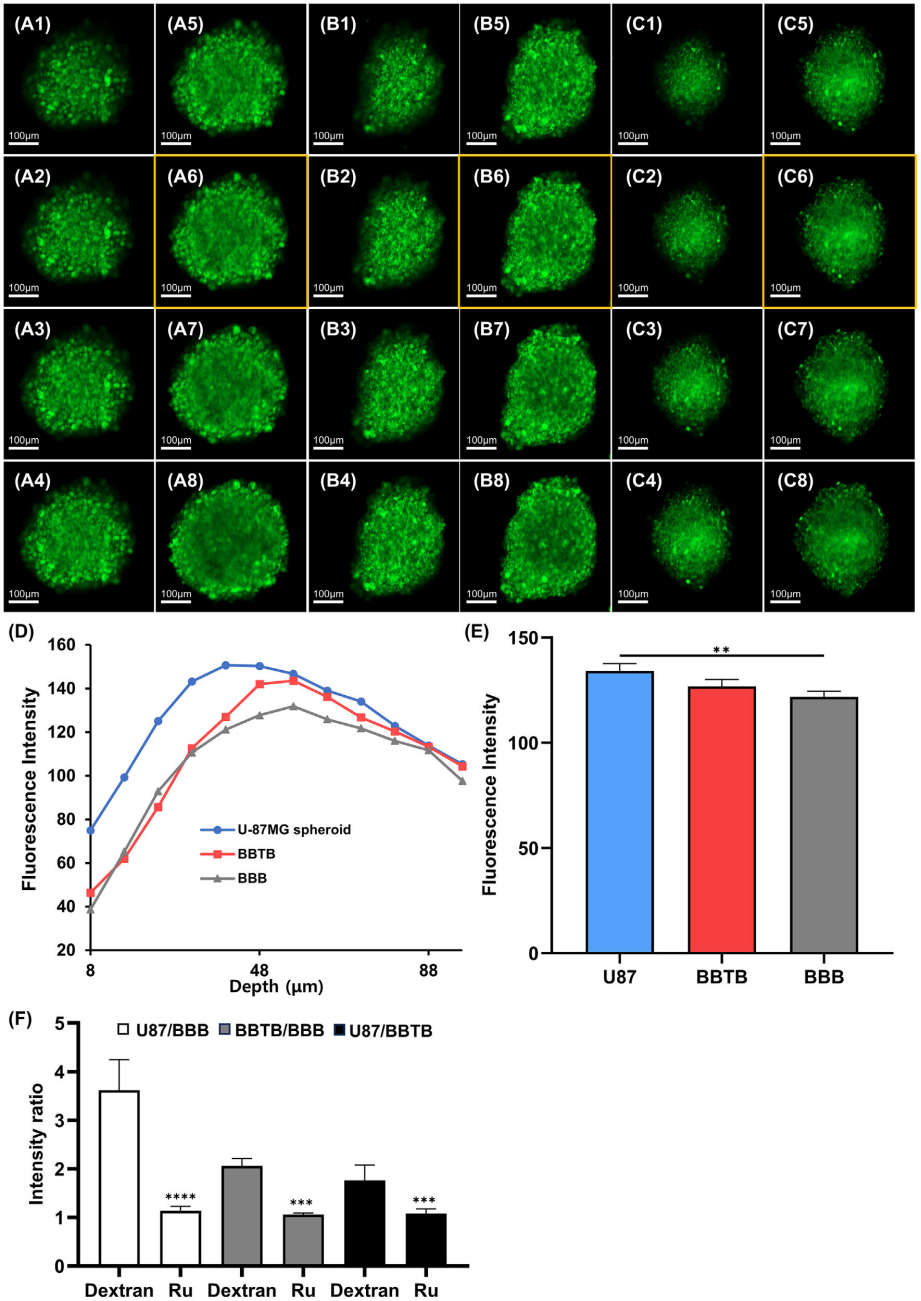
Depth‐resolved distribution of the Ru compound in U‐87 MG, BBTB, and BBB spheroids. (A1–A8, B1–B8, C1–C8) Serial cross‐sectional confocal images of Ru compound fluorescence acquired at multiple depths from U‐87 MG spheroids (A), BBTB spheroids (B), and BBB spheroids (C). (D) Depth‐resolved Ru compound fluorescence intensity profiles for each spheroid type, with fluorescence intensity (y‐axis) plotted against imaging depth (x‐axis, 8–96 µm). (E) Ru compound fluorescence intensity among the three spheroid types at 72 µm depth. (F) Fluorescence intensity ratio between spheroid groups (U‐87 MG/BBB, BBTB/BBB, and U‐87 MG/BBTB) for Dextran‐FITC and the Ru compound. Data are presented as mean ± SD of n = 3 separately formed and treated spheroids per group. Statistical significance was assessed by ordinary one‐way ANOVA with Tukey's multiple‐comparisons test. ***p < 0.001, ****p < 0.0001. Scale bar = 100 µm.

### 2.9 Cytotoxicity of Anticancer Agents in BBTB Spheroids

Existing BBB spheroids were fabricated using primary or immortalized astrocytes, pericytes, endothelial cells, and other BBB constituent cells. While these models are suitable for evaluating the permeability of fluorescent molecules, they pose challenges in assessing the BBB permeability of non‐fluorescent compounds. Furthermore, their utility in evaluating the cytotoxicity of anticancer drugs is limited, as cancer cells are not incorporated into the spheroid structures. In contrast, the core of the BBTB spheroid developed in this study comprises U‐87 MG cancer cells. Leveraging this unique configuration, the BBB permeability and anticancer efficacy of various agents were systematically investigated. In studies involving anticancer drugs targeting GBM, the effective concentration in U‐87 MG cells can reach several hundred micromolar (µM) [49]. Such elevated concentrations can compromise the reliability of cytotoxicity assessments when utilizing the BBTB spheroid. When comparing GBM‐specific agents with chemotherapeutic drugs at identical concentrations, cytotoxicity may not occur for GBM drugs due to subtherapeutic dosing. Conversely, administering chemotherapy agents at higher, cytotoxic doses may result in the death of constituent cells within the BBTB spheroid and disrupt BBB integrity. To mitigate these issues, the concentration range of anticancer agents was determined based on the IC_50_ concentrations in U‐87 MG cells.

Cancer cell death within the BBTB spheroids was visualized using a fluorescent probe. The anticancer efficacy of four agents—two that do not traverse the BBB (PTX and CIS), TMZ (penetrates the BBB), and the Ru compound—was assessed using the fabricated BBTB spheroid. Cross sections of the BBTB spheroids treated with four anticancer agents at a depth of 72 µm revealed that PTX and CIS did not differ significantly from the untreated control (Figure 11A). In contrast, the Ru compound and TMZ induced distinct cancer cell death signals. Importantly, these signals were absent in controls, confirming that they were not attributable to necrotic cores.

**FIGURE 11:**
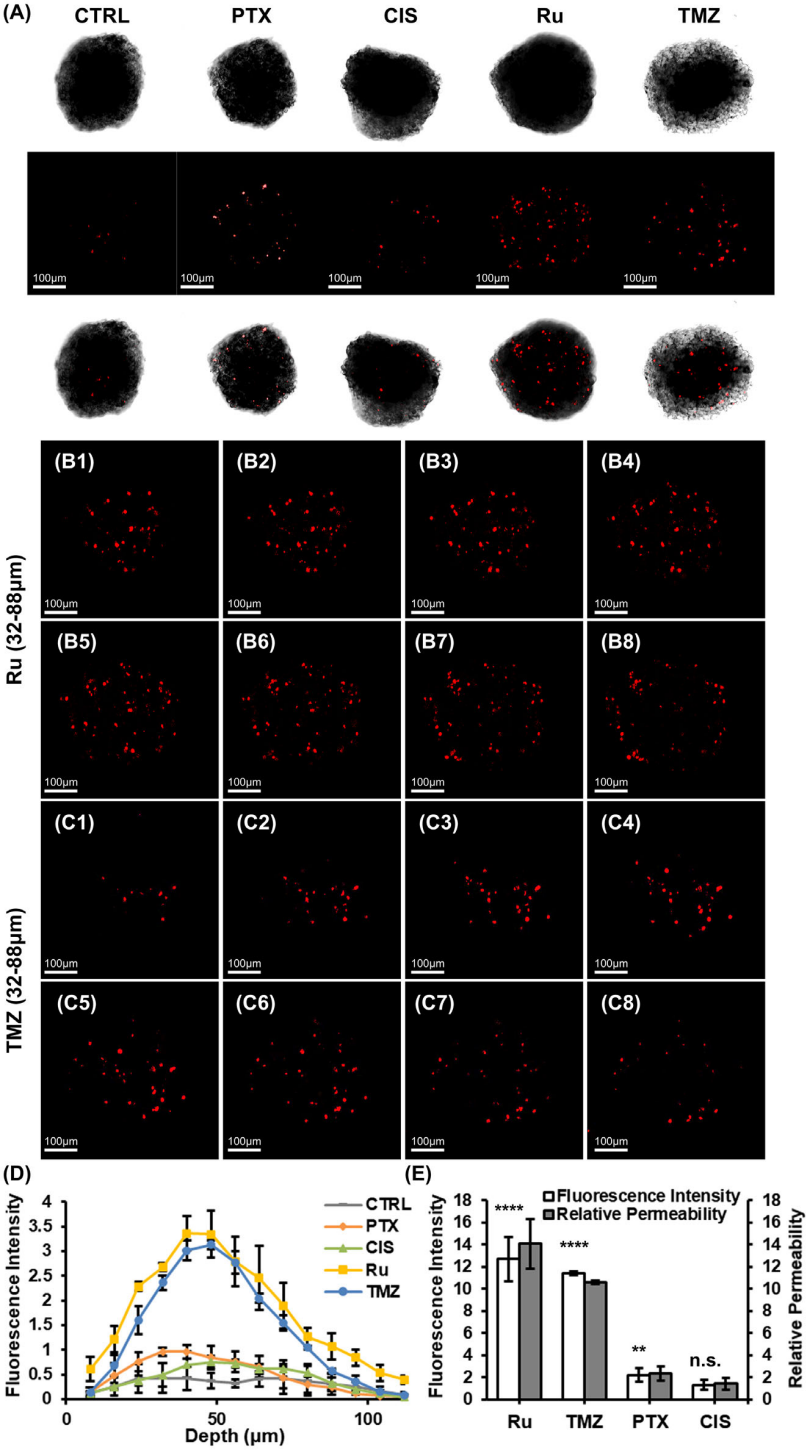
Anticancer response of test compounds in BBTB spheroids assessed by 7‐AAD staining. (A) Representative cross‐sectional confocal images of BBTB spheroids at a depth of 72 µm following incubation with each anticancer agent (control, PTX, CIS, Ru, and TMZ). Images show bright‐field (top), 7‐AAD fluorescence (middle), and merged channels (bottom). (B1–B8 and C1–C8) Serial cross‐sectional confocal images of 7‐AAD fluorescence acquired at depths of 32–88 µm (8 µm intervals) from BBTB spheroids treated with Ru (B) or TMZ (C). (D) Depth‐resolved 7‐AAD fluorescence intensity profiles of each treatment group, with fluorescence intensity (y‐axis) plotted against imaging depth (x‐axis). (E) Quantification of 7‐AAD fluorescence intensity (left y‐axis, open bars) and relative permeability index (right y‐axis, filled bars) for each treatment group, based on the summed 7‐AAD fluorescence intensity within the 32–64 µm depth range. PTX, paclitaxel; CIS, cisplatin; Ru, ruthenium compound; TMZ, temozolomide. Data are presented as mean ± SD of n = 3 separately formed and treated spheroids per group from a single independent experiment. Statistical significance was assessed by ordinary one‐way ANOVA followed by Tukey's multiple‐comparisons test, using a two‐sided alpha of 0.05. **p < 0.01, ****p < 0.0001; n.s., not significant. Analyses were performed using GraphPad Prism 11.0.2. Scale bar = 100 µm.

For the Ru and TMZ, multiple cell death images of cross sections were acquired at depths of 32–88 µm from the surface, at 8 µm intervals, corresponding to the region containing U‐87 MG cells (Figure 11B,C). Both showed an increasing trend in fluorescence signal up to 56–64 µm, followed by a sharp decline (Figure 11D), likely due to optical limitations imposed by the spheroid size as well as restricted penetration of Ru, TMZ, and probe. Analysis of cell death signal at depths of 32 to 88 µm showed that Ru exhibited slightly higher anticancer efficacy compared with TMZ. This difference was particularly evident in the bar graph of Figure 11E, which represents cancer cell death intensity of anticancer agents summed across depths of 32—64 µm.

To confirm that the 7‐AAD signal derives from the tumor population rather than from other constituents of the construct, the spheroids were assembled with CellTracker‐labeled U‐87 MG cells, treated with Ru, and stained with 7‐AAD, under the same conditions. The 7‐AAD signal was found to be confined to the labeled tumor core, with 91% ± 5.45% of 7‐AAD‐positive cells located within the labeled tumor volume, whereas the surrounding unlabeled layer showed little signal (Figure S5).

To accurately evaluate and compare the actual permeabilities of the Ru compound and TMZ within the BBTB spheroid, their treatment concentrations and IC_50_ concentrations were standardized against U‐87 MG cells in this model. This approach was necessary, as BBB permeability of anticancer drugs was determined by measuring cancer cell death in this study, which depends on both the treatment concentration and IC_50_ value of the tested anticancer drug. This relationship was expressed using the following equations:

$$\begin{array}{ccc} & & Number\;of\;cancer\;cell\;death\\ & & =K\times \frac{drug\;concentration\left(\mu M\right)\times BBB\;permeability}{IC_{50}\left(\mu M\right)} \end{array}$$

$$\begin{array}{ccc} & & Fluorescence\;intensity\;of\;dead\;cancer\;cell\\ & & =K^{\prime }\times \frac{drug\;concentration\left(\mu M\right)\times BBB\;permeability}{IC_{50}\left(\mu M\right)} \end{array}$$

where K and K′ are the proportionality constant related to cancer cell death in the BBTB spheroid. This equation can be expressed in terms of relative BBB permeability by eliminating the proportionality constant as follows:

$$Relative\;Permeability=\frac{\left(FI_{treated}-FI_{control}\right)\times IC_{50}\left(\mu M\right)}{Drug\;concentration\left(\mu M\right)}$$

where FI_treated_ represents the summed 7‐AAD fluorescence intensity in the defined U‐87 MG region following drug treatment, and FI_control_ is the corresponding baseline fluorescence of the untreated control. The Ru compound (IC_50_ = 22.3 µM, treatment = 20 µM; ΔFI = 12.7; IC_50_ ratio = 1.12) yielded a relative permeability of 14.2, whereas TMZ (IC_50_ = 223.1 µM, treatment = 240 µM; ΔFI = 11.4; IC_50_ ratio = 0.93) yielded a value of 10.6. Notably, although TMZ was administered at a concentration slightly exceeding its IC_50_ (ratio = 0.93), and the Ru compound was dosed marginally below its IC_50_ (ratio = 1.12), yet achieved a 1.34‐fold higher relative permeability than TMZ after normalization. In contrast, CIS (relative permeability = 1.4) and PTX (relative permeability = 2.4) exhibited markedly lower values, consistent with their established inability to penetrate the BBTB (Table 1).

**TABLE 1:** Relative permeability values for each compound in the BBTB spheroid.

Drug	IC_50_ (µM)	Drug Conc. (µM)	IC_50_ ratio	ΔFI	Relative Permeability
Ru	22.3	20	1.12	12.7	14.2
TMZ	223.1	240	0.93	11.4	10.6
PTX	5.3	5	1.06	2.2	2.4
CIS	9.5	9	1.06	1.3	1.4

These results demonstrate that even when the Ru compound was administered at a concentration slightly below its IC_50_, it provided greater anticancer efficacy per unit dose than TMZ. Hence, the Ru compound may possess enhanced BBTB penetration and robust anticancer efficacy against U‐87 MG glioblastoma cells in the BBTB spheroid model.

### 2.10 Validation of the BBTB Spheroid Platform Using Patient‐Derived GBM Cells

Given the heterogeneity of patient glioblastoma, we examined whether the platform could be reconstituted with patient‐derived GBM cells. BBTB spheroids were assembled using two patient‐derived cell lines, SNU‐4210 and SNU‐5026, in place of U‐87 MG cells, with hCMEC/D3 cells and HBVPs at the same 1:1:1 ratio. In both cases, spheroids formed spontaneously, and CellTracker labeling of each cell type confirmed the same layered organization observed for the U‐87 MG‐based model, with hCMEC/D3 cells in the outermost layer, HBVPs forming an intermediate layer, and the patient‐derived glioblastoma cells comprising the core (Figure S6).

Day‐3 spheroids were treated with the Ru compound (20 µM) or TMZ (240 µM) for 48 h, and cell death was assessed by 7‐AAD staining with confocal imaging at 8 µm intervals. In both patient‐derived models, the Ru compound produced substantially greater 7‐AAD signal than TMZ (Figure 12). Summed fluorescence intensity across depths of 32–64 µm was 24.10 ± 5.84 for Ru versus 10.57 ± 3.77 for TMZ in SNU‐5026 spheroids, and 25.74 ± 5.86 versus 12.44 ± 3.50 in SNU‐4210 spheroids, corresponding to 2.3‐fold and 2.1‐fold higher signal for the Ru compound, respectively. Vehicle‐treated controls showed minimal signal throughout, and the depth profiles peaked at approximately 40 µm in both cell lines, consistent with the distribution observed in the U‐87 MG‐based model. These results indicate that the platform is not restricted to a single established cell line and can be applied to patient‐derived GBM cells.

**FIGURE 12:**
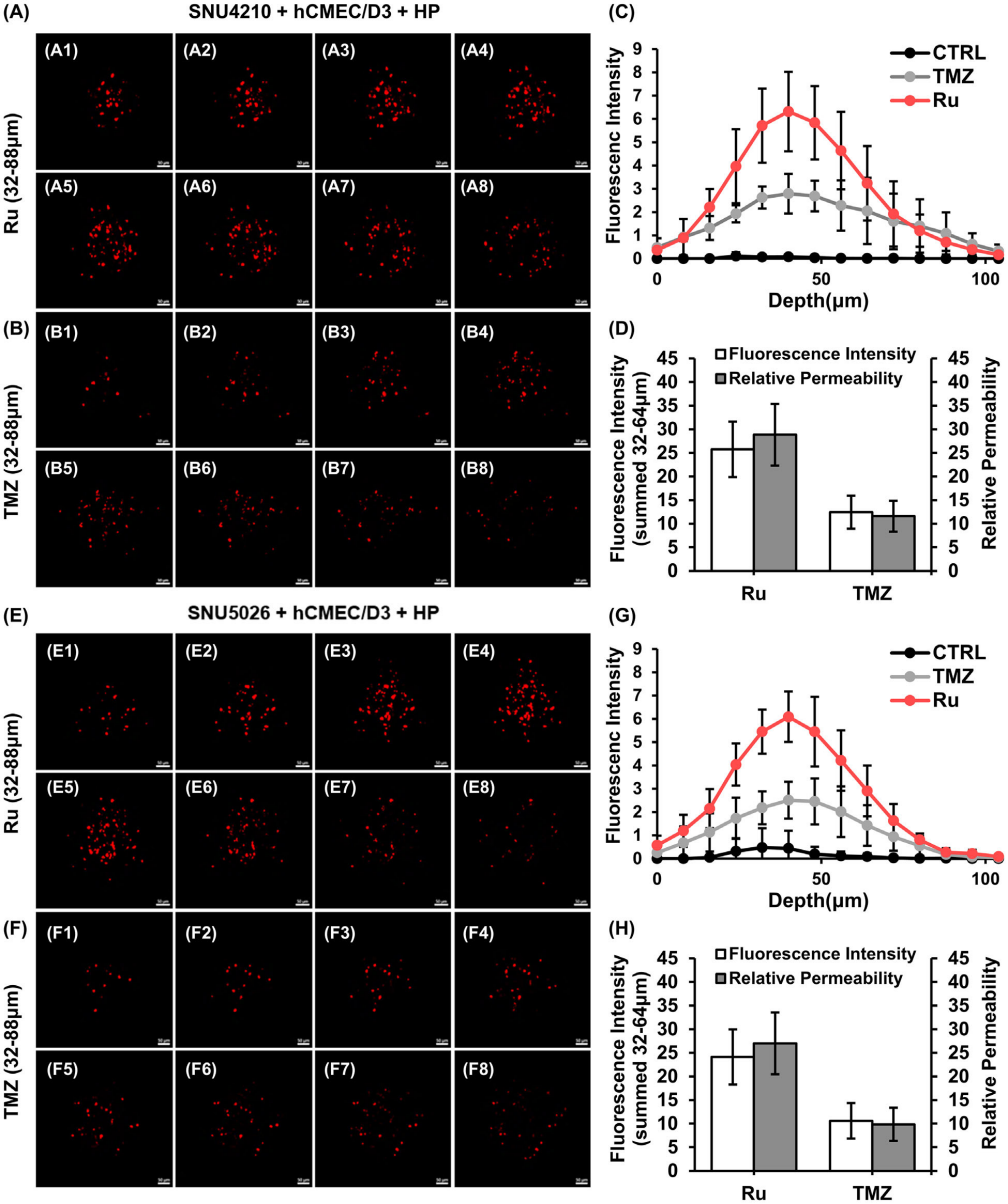
Validation of the BBTB spheroid platform using patient‐derived glioblastoma cell lines. BBTB spheroids were assembled with the patient‐derived glioblastoma cell lines SNU‐4210 (A–D) or SNU‐5026 (E–H) in place of U‐87 MG cells, together with hCMEC/D3 endothelial cells and HBVPs at a 1:1:1 ratio. Day‐3 spheroids were treated with the Ru compound (20 µM) or temozolomide (TMZ, 240 µM) for 48 h, after which cell death was assessed by 7‐AAD staining and confocal imaging at 8 µm intervals. (A,B) Serial cross‐sectional confocal images of 7‐AAD fluorescence in SNU‐4210 spheroids treated with the Ru compound (A) or TMZ (B), acquired at depths of 32–88 µm from the spheroid surface. (C) Depth profiles of 7‐AAD fluorescence intensity in SNU‐4210 spheroids. (D) 7‐AAD fluorescence intensity and relative permeability index summed across depths of 32–64 µm in SNU‐4210 spheroids. (E,F) Corresponding confocal images for SNU‐5026 spheroids treated with the Ru compound (E) or TMZ (F). (G) Depth profiles for SNU‐5026 spheroids. (H) Summed 7‐AAD fluorescence intensity and relative permeability index for SNU‐5026 spheroids. Vehicle‐treated control spheroids showed negligible 7‐AAD signal and are shown in the depth profiles only. The relative permeability index was derived from the summed fluorescence intensity by normalization to the corresponding drug concentration and IC_50_, and is presented for comparison only. Scale bars = 50 µm. Data are presented as mean ± SD of n = 3 separately formed and treated spheroids per group from a single independent experiment.

### 2.11 Transendothelial Transport and P‐gp Substrate Profiling Across an hCMEC/D3 Monolayer

To identify effective concentrations that preserve endothelial barrier integrity yet retain GBM cell cytotoxicity, viability assays were performed using CellTiter‐Glo for hCMEC/D3 cells (Figure 13A1–A4) and the MTT assay for U‐87 MG cells (Figure 13B1–B3). hCMEC/D3 viability was retained at 96.86% following treatment with the Ru compound at 30 µM, but declined to 64.89% at 50 µM; accordingly, 30 µM was selected as the highest safe dose, ensuring no cell death. The Ru compound demonstrated cytotoxicity toward U‐87 MG cells starting at 10 µM (cell viability: 82.75%). Selecting a concentration of 20 µM, within the safe range for hCMEC/D3 cells, offered a clear therapeutic window.

**FIGURE 13:**
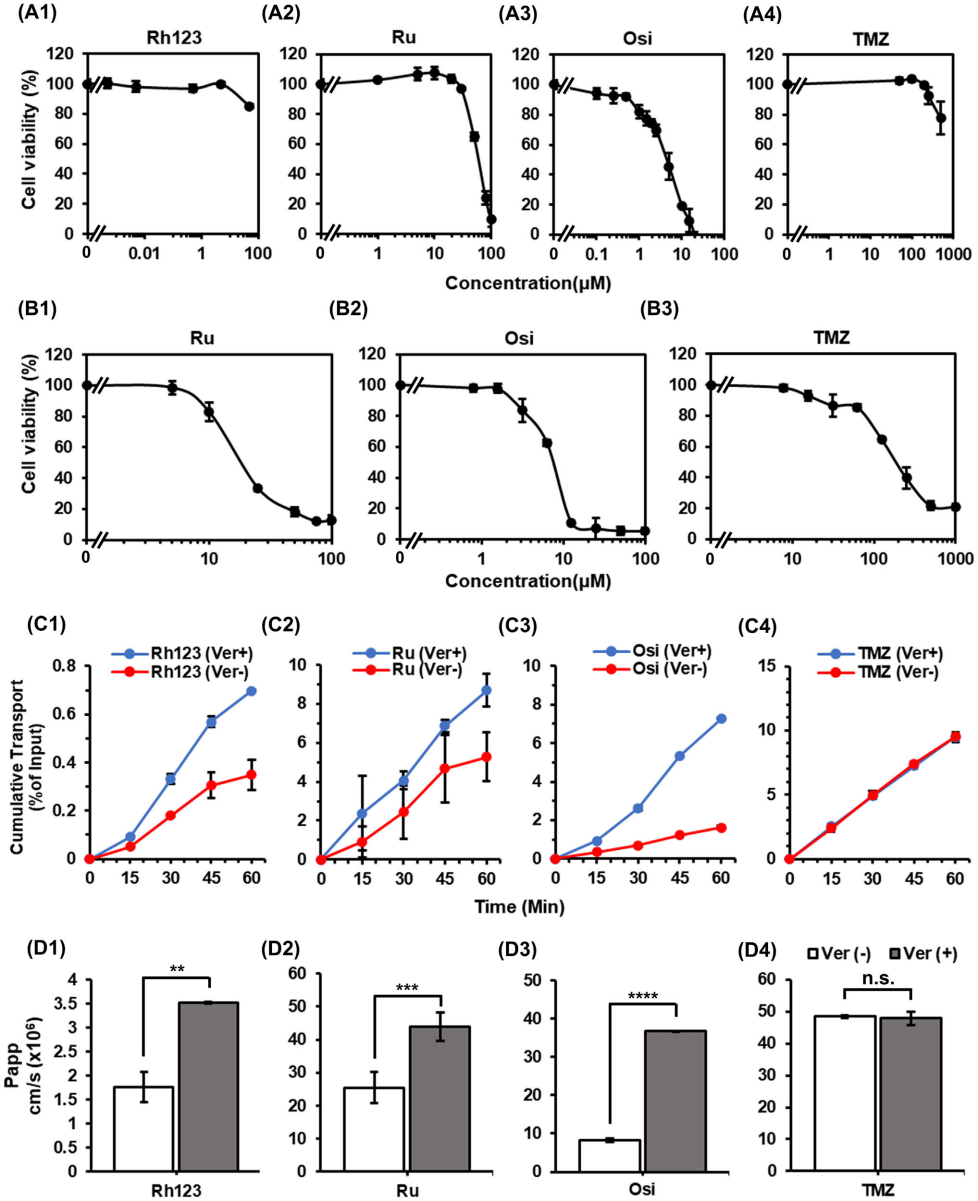
Cytotoxicity profiling and Transwell permeability assessment of test compounds across hCMEC/D3 and U‐87 MG cell models. (A1–A4) Cell viability of hCMEC/D3 human cerebral microvascular endothelial cells following 24 h treatment with Rh123 (A1), Ru (A2), Osi (A3), or TMZ (A4), assessed by CellTiter‐Glo luminescent cell viability assay. (B1–B3) Cell viability of U‐87 MG glioblastoma cells following 48 h treatment with Ru (B1), Osi (B2), or TMZ (B3), assessed by MTT assay. (C1–C4) Cumulative apical‐to‐basolateral transport profiles of Rh123 (C1), Ru (C2), Osi (C3), and TMZ (C4) across hCMEC/D3 monolayers in the presence (Ver+) or absence (Ver−) of the P‐glycoprotein inhibitor verapamil (Ver, 100 µM), measured at 15‐min intervals over 60 min. (D1–D4) Apparent permeability coefficients (P_app_) of Rh123 (D1), Ru (D2), Osi (D3), and TMZ (D4) in the presence (Ver+) or absence (Ver−) of verapamil. **p < 0.01, ***p < 0.001, ****p < 0.0001; n.s., not significant. Rh123, Rhodamine‐123; Ru, ruthenium compound; Osi, osimertinib; TMZ, temozolomide; Ver, verapamil. Data in (A1–A4) and (B1–B3) are presented as mean ± SD of n = 3 replicate wells per concentration seeded in parallel; data in (C1–C4) and (D1–D4) are presented as mean ± SD of n = 3 Transwell inserts seeded in parallel per condition. Statistical significance was assessed by ordinary one‐way ANOVA followed by Tukey's multiple‐comparisons test, using a two‐sided alpha of 0.05. Analyses were performed using GraphPad Prism 11.0.2.

TMZ demonstrated distinct cytotoxicity profiles: hCMEC/D3 cell viability was 92.67% at 250 µM and decreased to 77.64% at 500 µM, whereas U‐87 MG cytotoxicity was observed from 31.25 µM (86.64%) and intensified at 125 µM (64.68%). Thus, 240 µM TMZ was selected as cytotoxic for U‐87 MG cells, without affecting hCMEC/D3 cell viability. These findings suggest that the Ru compound and TMZ have suitable selectivity indices for BBTB‐targeted therapy. Conversely, osimertinib demonstrated overlapping cytotoxicity between U‐87 MG and hCMEC/D3 cells. U‐87 MG viability after osimertinib treatment decreased to 83.62% at 3.125 µM (estimated IC_50_: 8.5 µM), but was nearly 100% at 0.5 µM (Figure 13B2). However, in hCMEC/D3 cells, osimertinib led to 92.23% viability at 0.5 µM and 69.68% at 2.5 µM (Figure 13A3). For BBTB spheroid treatment, 0.5 µM osimertinib was selected, as it was the highest concentration with minimal impact on hCMEC/D3 viability. These findings suggest that the concentration range required for osimertinib to exert cytotoxicity against GBM cells substantially overlaps with that required to induce toxicity in hCMEC/D3 endothelial cells, making its therapeutic window insufficient for BBTB spheroids.

The Transwell system used hCMEC/D3 monolayers cultured on 0.4‐µm‐pore inserts for 7 days. Monolayer integrity was measured using Lucifer Yellow (LY; 100 µM), a marker for paracellular integrity. The P_app_ (LY) was 1.53 × 10^−^^6^ cm/s, confirming sufficient barrier integrity for comparative drug permeability assessment in this study (Figure S7). Apical‐to‐basolateral transport (A→B) was analyzed for the Ru compound (20 µM), TMZ (240 µM), and osimertinib (0.5 µM); Rhodamine‐123 (Rh123, 5 µM) served as a positive control for P‐gp‐mediated efflux. Parallel experiments were conducted with and without the P‐gp inhibitor verapamil (100 µM) to evaluate P‐gp's role in this transport. Drug transport across the hCMEC/D3 monolayer was quantified by sequentially transferring the Transwell insert into fresh basolateral wells at 15‐min intervals over a total of 60 min (0, 15, 30, 45, and 60 min), enabling interval‐specific permeation measurement that minimizes back‐diffusion errors.

Osimertinib and TMZ were quantified in basolateral samples via LC–MS/MS with specific SRM transitions (*m*/*z* 500.267 → 71.984 for osimertinib; *m*/*z* 194.850 → 137.933 for TMZ), while the Ru compound and Rh123 were analyzed using fluorescence spectrophotometry. To prevent matrix interference, calibration standards were matrix‐matched with basolateral samples, yielding high coefficients of determination (*R* ^2^ = 0.9953 for osimertinib and *R* ^2^ = 0.9983 for TMZ), thus confirming reliable quantification across the established concentration ranges. The specificity of each LC–MS/MS method was validated by comparing retention times of sample peaks with those of reference standards.

The cumulative percentage transport–accumulation profiles for all the compounds are shown in Figure 13C1–C4. Rhodamine‐123 (Rh123, 5 µM), which served as the positive control for P‐gp‐mediated efflux, exhibited markedly lower cumulative transport in the verapamil‐free condition (Ver−) than in the verapamil‐treated group (Ver+). This confirmed functional P‐gp activity in the hCMEC/D3 monolayer. The Ru compound showed a modest but nearly consistent difference in cumulative transport between Ver+ and Ver− treatments across all time points. TMZ displayed nearly identical cumulative transport curves for both conditions. Osimertinib displayed a marked increase in cumulative transport differences over time between Ver+ and Ver− treatments. This suggested that strong P‐gp‐mediated efflux of osimertinib occurred in hCMEC/D3 cells.

The apparent permeability coefficients (P_app_) calculated from the 60‐min basolateral accumulation data are presented in Figure 13D1–D4. To quantify the extent of P‐gp involvement, the efflux ratio was defined as the fold‐change in P_app_ (Ver+) relative to P_a__pp_ (Ver−). For Rh123, P_app_ (Ver+) was 3.52 × 10^−^^6^ cm/s and P_app_ (Ver−) was 1.76 × 10^−^^6^ cm/s, yielding an efflux ratio of approximately 2.0‐fold (Figure 13D1–D4). This increase upon P‐gp inhibition is consistent with established literature values for Rh123 in hCMEC/D3 monolayers, confirming that the P‐gp efflux system was functionally well established in the present model and validating Rh123 as a reliable positive control for subsequent P‐gp substrate profiling. For the Ru compound, P_app_ (Ver+) was 43.86 × 10^−^^6^ cm/s and P_app_ (Ver−) was 25.51 × 10^−^^6^ cm/s, corresponding to an efflux ratio of 1.7‐fold. This indicated that P‐gp partially restricted Ru transport and that transcellular passive diffusion was a partial route for its penetration across the BBB. For TMZ, the P_app_ (Ver+) was 47.91 × 10^−^^6^ cm/s and P_app_ (Ver−) was 48.19 × 10^−^^6^ cm/s, yielding an efflux ratio of 0.99‐fold—essentially unchanged between the two conditions—confirming that TMZ is not a P‐gp substrate and crosses the endothelial monolayer exclusively via P‐gp‐independent passive diffusion. For osimertinib, the P_app_ (Ver+) was 36.67 × 10^−^^6^ cm/s and P_app_ (Ver−) was 8.23 × 10^−^^6^ cm/s, yielding an efflux ratio of approximately 4.4‐fold. The pronounced increase upon verapamil treatment indicates that P‐gp‐mediated efflux substantially limits the transport of osimertinib across the hCMEC/D3 monolayer. P‐gp acts by reducing the intracellular retention of the compound, and the limitation in transport follows from this.

Cumulatively, the Transwell permeability data support the mechanistic validation for 7‐AAD‐based intact cancer cell death imaging in BBTB spheroids and position the Ru compound as a promising candidate for BBTB‐permeable therapeutics. While TMZ—a non‐P‐gp substrate—serves as the reference standard for BBTB penetration, the Ru compound achieved a 33.9% higher relative permeability in the BBTB spheroid despite being administered at a concentration below its IC_50_. The Transwell data further clarified that the Ru compound crosses the BBB endothelium partially via transcellular passive diffusion, with less P‐gp‐mediated efflux (1.7‐fold) than osimertinib (4.4‐fold). The absence of a therapeutic window for osimertinib in hCMEC/D3 cells and its strong P‐gp efflux collectively disqualify it as a viable BBTB‐penetrant GBM therapeutic. In contrast, the Ru compound's selective toxicity against U‐87 MG cells at concentrations tolerable for hCMEC/D3 cells strongly supports its further development as a potential next‐generation anti‐GBM therapeutic candidate.

### 2.12 Cytotoxicity of Anticancer Agents in BBTB Spheroids Using FACS

To confirm the validity of the cytotoxicity assays, cell death in BBTB spheroids treated with anticancer agents was assessed by FACS. U‐87 MG cells were first labeled with the CMFDA cell tracker before spheroid formation, enabling their distinction from other cell types. The anticancer activities of CIS, PTX, the Ru compound, and TMZ were evaluated in BBTB spheroids containing these CMFDA‐stained U‐87MG cells. After treatment with each agent, cells were stained with 7‐AAD, and flow cytometry was used to analyze cell death. Gating on CMFDA‐positive cells identified the U‐87 MG population (Figure 14A). The dead cell population (M1) was defined based on 7‐AAD single‐stained controls (Figure 14B). Consistent with the previous results, control samples exhibited approximately 1.6% cell death. Treatment with CIS and PTX induced 2.7% and 3.5% cell death, respectively, with no significant differences compared with the control. In contrast, the Ru compound and TMZ treatments induced 21.7% and 19.9% cell death, respectively, higher than the control. The ratios of U‐87 MG cell death induced by the Ru compound and TMZ exceeded those induced by CIS and PTX. These outcomes aligned with the results of image‐based analyses, supporting the reliability of the imaging method for quantitative cytotoxicity assessment.

**FIGURE 14:**
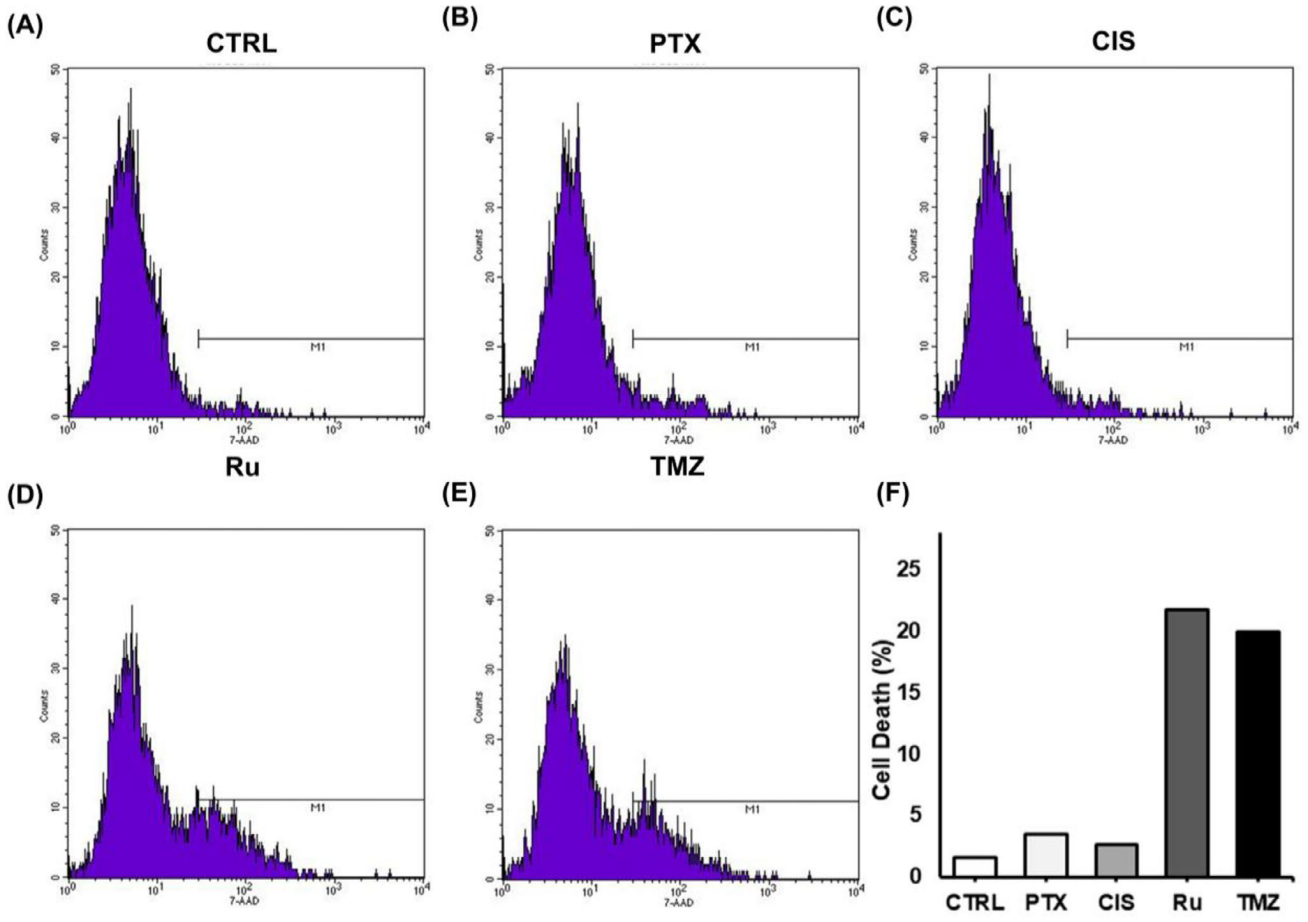
Cross‐validation of tumor‐cell death in BBTB spheroids by flow cytometry. (A–E) Representative flow cytometry histograms of 7‐AAD fluorescence in U‐87 MG cells dissociated from BBTB spheroids following treatment with control (A), PTX (B), CIS (C), Ru (D), or TMZ (E). (F) Quantification of cell death (%) expressed as the ratio of 7‐AAD‐positive U‐87 MG cells to the total cell population across treatment groups. PTX, paclitaxel; CIS, cisplatin; Ru, ruthenium compound; TMZ, temozolomide. Values are from a single preparation, with n = 20 spheroids pooled per condition to provide sufficient cells for acquisition; bars therefore show the measured percentage for each condition and no statistical comparison was performed. Histograms are representative of that preparation.

## 3 Discussion

GBM is the most aggressive intracranial malignancy that originates primarily from astrocytes. The BBB​​ blocks the movement of molecules inside and outside the brain. Through both direct and indirect interactions, GBM affects BBB constituent cells, such as pericytes, and even exploits them for proliferation via cytoplasmic mixing. Consequently, BBTB within TME exhibits characteristics such as relatively leaky and heterogeneous permeability. The BBTB represents a major obstacle in treating GBM, as it limits the transport of anticancer drugs from blood vessels into tumor. Although imaging techniques such as MRI can capture the overall movement of anticancer drugs into brain tumors, directly assessing cancer cell death induced by anticancer agents at the single‐cell level remains extremely challenging. This necessitates the need for an in vivo mimic BBTB model that closely replicate the actual in vivo TME. In constructing a brain tumor spheroid that forms a BBB, an ideal model must satisfy several criteria: direct interactions among the three constituent cells, sufficient expression of TJ proteins to establish the BBB, and unlike the BBB spheroid, cytoplasmic mixing between GBMs and pericytes in the BBTB spheroid. These conditions exist in actual brain tumor models, and replicating these conditions is a major challenge in fabricating BBTB spheroids.

Unlike the spheroid production approach in this study, which simulates a brain tumor model based on direct interactions among three cell types (vascular endothelial cells, pericytes, and U‐87 MG cells), many studies have developed scaffold‐based BBB brain tumor models using microfluidics systems (e.g., a tubular BBTB microfluidic chip model). However, in these scaffold‐based models, direct interactions among vascular endothelial cells, astrocytes, and U‐87 MG cells are limited, as the scaffold separates the inner and outer compartments. In contrast, the BBTB spheroid model developed in this study successfully achieves direct cell–cell contact. The cytoplasmic mixing occurring between pericytes and U‐87 MG is clearly observed at the BBTB edge (Figure 3). This cytoplasmic mixing, a hallmark of actual brain tumors, contributes to weakening of the BBB in tumors compared with normal brain tissue. The dextran permeability pattern in this study demonstrated this characteristic behavior inherent to GBMs.

Beyond cytoplasmic mixing, the expression of TJ proteins and P‐gp is crucial for evaluating the integrity of the BBTB spheroid models. P‐gp, encoded by the *ABCB1* gene, is a well‐characterized member of the human ABC transporter family and plays a critical role in exporting drugs and toxic substances from cells. Vascular endothelial cells at the BBB rely on high‐affinity interactions facilitated by TJ proteins—such as ZO‐1, occludin, and claudin‐5—to prevent substance penetration. Accordingly, the expression levels of these proteins were quantitatively evaluated in BBTB spheroids using single‐cell imaging. This confirmed successful BBTB spheroid fabrication with over 90% of observed vascular endothelial cells expressing all four proteins. Furthermore, dextran permeability assays demonstrated increased permeability in BBTB spheroids, which exhibited cytoplasmic mixing and TJ and P‐gp protein expression, relative to BBB spheroids. These findings substantiate the model's capability to simulate the distinct characteristics of brain tumors with high fidelity.

This study introduces an innovative BBTB spheroid model designed to simulate brain tumors and assess the anticancer efficacy of potential therapeutic candidates within intact spheroids, eliminating the need for dissociation to the single‐cell level. While traditional methods exist for quantifying cancer cell death induced by anticancer compounds, these approaches are limited by the inability of conventional probes to traverse the BBTB barrier and penetrate spheroids, thereby preventing direct observation of cell death. Common reagents, such as Annexin‐V and PI are limited in their utility due to poor penetration associated with the structural characteristics of BBB spheroids. Consequently, conventional evaluation requires enzymatic dissociation (e.g., using accutase), followed by multiple centrifugation and washing steps before FACS analysis, which inevitably leads to cell loss.

In contrast, when using a cancer cell death probe that crosses the BBB and reaches the spheroid interior, tumor cell death can be evaluated without disrupting spheroid integrity or requiring extensive staining or washing procedures. In this study, application of the 7‐AAD probe enabled direct visualization of anticancer drug‐induced cell death within intact BBTB spheroids. The findings demonstrate that 7‐AAD effectively penetrates the BBTB barrier and enables detection of apoptotic cell death at the single‐cell level following treatment with anticancer agents.

This method offers a robust alternative to the previously established approach of measuring tumor size reduction, which is often confounded by variability in spheroid dimensions and does not reliably reflect cell death or treatment efficacy. Instead, the proposed technique facilitates precise, quantitative analysis of anticancer effects using in situ single‐cell statistics [50]. Using the BBTB spheroid model, we evaluated the BBB penetration and anticancer efficacy of CIS, PTX, TMZ, osimertinib, and a Ru compound. As expected, CIS and PTX exhibited minimal efficacy due to poor BBB permeability, whereas TMZ and the Ru compound—both possessing BBB permeability—demonstrated clear anticancer effects. These findings underscore the value of the proposed method for accurately assessing brain tumor therapies and their ability to induce tumor cell death within the physiologically relevant context of intact BBTB spheroids.

Within the BBB, small‐molecule compounds primarily enter via transcellular transport across the vascular endothelial cells. However, brain vascular endothelial cells actively excrete foreign substances—including low‐molecular‐weight compounds—through ABC transporters such as P‐gp, thereby reinforcing the integrity of the BBB and protecting neural tissue. Paracellular diffusion, which proceeds through the intercellular spaces between adjacent endothelial cells, is tightly regulated by TJs. The TJ proteins effectively block the paracellular space, physically preventing most substances from entering the brain through this route and further strengthening the BBB's barrier function. Thus, only select small molecules can passively diffuse through these narrow intercellular gaps.

While significant progress has been made in developing small‐molecule anticancer agents targeting brain tumors, clinical translation remains challenging. This difficulty largely arises because these compounds must penetrate vascular endothelial cells containing ABC transporters via transcellular routes—even when their in vitro cytotoxicity against brain tumor cells is high. Most anticancer agents are P‐gp substrates and thus have limited ability to penetrate vascular endothelial cells, cross the BBB, and exert anticancer effects against brain tumors.

Findings from this study confirmed this trend: anticancer agents with relatively high molecular weights, such as PTX (853.9 g/mol), could not effectively cross the BBTB and demonstrated negligible penetration and cytotoxicity toward U‐87 MG cells. In contrast, TMZ, with a lower molecular weight (194.15 g/mol) and documented BBB permeability (CSF/plasma ratio of ∼20%), exhibited greater permeability than controls. Notably, osimertinib showed a substantial P_a__p__p_ efflux ratio (4.4‐fold) upon Verapamil+/Verapamil− treatment, although it was not designed to act as a P‐gp substrate. Moreover, osimertinib failed to demonstrate therapeutic efficacy against U‐87 MG cells at the highest concentration tested, which was not toxic to hCMEC/D3 cells. These observations suggest that osimertinib may not be an effective therapeutic candidate against U‐87 MG cells.

The criterion for permeabilization in this investigation was defined by assessing cell mortality following permeabilization procedures and measuring anticancer efficacy against U87 MG cells. Variations in cancer cell death rates induced by the tested drugs were attributed to differences in their IC_50_ values for U‐87 MG cells and their respective BBTB permeabilities. This study introduces a new metric—relative BBB permeability—derived from correlations between treatment concentrations and IC_50_ values of anticancer drugs, to evaluate the actual BBB permeabilities of tested compounds within BBTB spheroids containing U‐87 MG cells. Among the anticancer drugs, TMZ demonstrated permeability levels sufficient to be considered permeable through the BBTB. Conversely, CIS and PTX showed notably poor relative permeability values. CIS, a platinum‐based agent with low solubility and a molecular weight of 301.1 g/mol, did not traverse the BBB within the BBTB spheroid model, likely due to its hydrophilic nature; the BBB typically restricts such substances while permitting lipophilic molecules. Similarly, PTX, which is highly lipophilic and relatively large (853.9 g/mol), failed to demonstrate transcellular transport in BBTB spheroids. Importantly, BBB endothelial cells express efflux transporters, including P‐gp, which actively excrete drugs from brain tissue. The observation that CIS and PTX did not penetrate the BBB affirmed the successful formation of an effective barrier in the fabricated BBTB spheroid.

In this study, the anticancer drugs can be categorized into those that do not exhibit blood‐brain barrier (BBB) permeability and those that do, such as Temozolomide and Ru. For the numerous anticancer drugs that did not induce observable cancer cell death in BBTB spheroids, a scenario may arise where the drugs successfully permeated biological membranes but failed to elicit sufficient cytotoxicity to cause detectable cell death. In such cases, rather than implying a complete lack of permeability, it can be inferred that the drugs did cross the biological membranes, albeit to an extent that made the resulting cancer cell death difficult to measure. In light of this phenomenon, this study clarifies that the BBTB spheroid system did not measure absolute permeability; rather, it measured the permeability of certain anticancer drugs on a relative basis—specifically, in comparison to the other anticancer drugs being tested. It is also worth noting that for many anticancer drugs that do not exhibit BBB permeability—making it difficult to achieve an anticancer effect—the drugs may not be afforded the sufficient exposure time required for each of them. In this study, a 48‐hour exposure time was used to assess BBTB penetration and the induction of cancer cell death by the anticancer drugs. In previous research, our research team determined the extent of cancer cell death expressed as EC50 values using various tumor spheroids following this 48‐hour exposure. Typically, most anticancer drugs successfully induced cancer cell death in tumor spheroids within this timeframe. In the current study, temozolomide and Ru compounds induced sufficient cancer cell death for observation. Taking these factors into account, we measured BBB permeability in relative rather than absolute terms. 3D co‐culture systems yield values that differ from those of 2D monocultures. This is due to substantial differences in ECM composition and physical diffusion rates between 3D spheroids and 2D cultures. It was previously reported that EC50 values in 3D spheroids are approximately 2 to 3 times higher than in 2D monocultures across various tumor spheroid models [9]. Crucially, this increase follows a similar trend across the anticancer drugs tested. Therefore, since this study measures permeability in relative rather than absolute terms, it is reasonable to assume that this trend of relative increase is effectively reflected.

In this study, two considerations played a role in the selection of Ru as the test anticancer compound for BBB penetration. First of all, compared to other anticancer compounds tested in this study, the synthetic Ru compound possesses a different mode of action for its anticancer activity. Most tested anticancer compounds target DNA. On the other hand, the anticancer activity of Ru is based on ROS generation. Overcoming the blood‐brain barrier (BBB) involves two main mechanisms: transcellular transport and paracellular transport. P‐glycoprotein expressed on cerebrovascular endothelial cells actively exports compounds that have entered the cells, thereby preventing them from crossing the BBB. Among the compounds tested here, temozolomide was the only one that is not a P‐glycoprotein substrate. Ru is also not entirely free from this mechanism. On the other hand, the paracellular transport of anticancer drugs is restricted by the robust tight junction proteins formed in brain vascular endothelial cells. Unlike the other DNA‐targeting anticancer agents, the Ru compound was selected in this study based on the strong possibility that it could influence the expression of these tight junction proteins via ROS. Another notable property of the Ru compound is its intrinsic fluorescence. In the novel BBTB spheroid model proposed in this study, this fluorescence offers the advantage of enabling on‐site monitoring of the agent's distribution within the spheroid—provided it successfully crosses the BBB—and the resulting cancer cell death.

Notably, the Ru compound exerts anticancer effects via ROS generation and has a molecular weight of 871.9 g/mol. Despite its relatively high molecular weight, the Ru compound exhibited greater permeability through the BBTB than TMZ. Investigations into this phenomenon revealed that Ru acts, in part, as a P‐gp substrate but still achieves superior penetration. Treatment with the Ru compound activated a‐SMA and F‐actin, markers of epithelial–mesenchymal transition (EMT) in pericytes, indicating a loss of intercellular adhesion and polarity, as well as transformation into highly mobile mesenchymal cells. This transition undermines the structural integrity of the BBTB.

Furthermore, increased expression of MMP‐9, an enzyme involved in ECM degradation and thus in the maintenance of epithelial rigidity and BBB integrity, was observed in vascular endothelial cells and astrocytes following treatment with the Ru compound. This further suggests that Ru compromises BBTB integrity. Additionally, Ru exposure reduced tight junction protein levels, thereby enhancing barrier permeability. ET‐1, a known vasoconstrictor whose expression is elevated under oxidative stress, was also significantly upregulated in Ru‐treated astrocytes, as confirmed by ELISA assays. This vasoconstrictive response may further facilitate increased BBTB permeability. MMP‐9 and ET‐1, however, are pleiotropic mediators rather than barrier‐specific biomarkers, and their broader roles in tumor biology warrant consideration. In addition to remodeling tight‐junction and extracellular‐matrix components, MMP‐9 has been implicated in local GBM infiltration and tumor‐associated vascular remodeling [51]. Endothelin signaling has likewise been associated with glioma‐cell survival, migration, MMP‐9 expression, and microenvironment‐mediated treatment resistance [52, 53]. Barrier opening is likewise not a phenomenon unique to the Ru compound: ROS‐mediated tight junction loosening has been reported in brain endothelial cells exposed to platinum‐based agents in current use and implicated in their central nervous system toxicity [54], indicating that the ROS‐mediated changes of MMP‐9 and ET‐1 observed in this study fall within a mechanism shared by clinically established drugs. Enhanced compound passage arising from weakening of the BBB tight junction is therefore mechanistically distinct from favorable intrinsic BBTB penetration. This distinction, along with the in vivo balance between delivery to tumor and neurovascular safety, will require further evaluation to investigate the correlation between the barrier damage and dose‐limiting factor.

Collectively, these findings suggest that the Ru compound reaches the tumor compartment through ROS‐mediated mechanisms, supporting further evaluation as a potential therapeutic candidate for brain tumors.

## 4 Conclusions

A BBTB spheroid model was successfully developed, achieving cytoplasmic mixing between pericytes and U‐87 MG cells. TJ proteins—including ZO‐1, occludin, and claudin‐5—which are essential for maintaining BBB integrity in brain tumors, were clearly expressed in the BBTB spheroids. The anticancer efficacy of the test compounds was evaluated using in situ cell death imaging of U‐87 MG cells incorporated into the constructed BBTB model. TMZ, a lipophilic compound with low molecular weight, effectively induced U‐87 MG cell death by traversing the BBTB via passive transcellular diffusion. In contrast, CIS, despite its relatively low molecular weight, its hydrophilic nature prevented penetration of the BBTB. Similarly, lipophilic PTX with a higher molecular weight did not penetrate the BBTB. The synthetic Ru compound, an ROS generator, exerted various effects on the BBTB that may facilitate its penetration through paracellular and partial transcellular transport. Accordingly, the Ru compound demonstrated superior anticancer efficacy against U‐87 MG cells compared to TMZ, despite partially serving as a substrate for P‐gp.

## 5 Experimental Section

### 5.1 Cell Culture and Media Composition

U‐87 MG glioblastoma cells (KCLB, Korea; RRID: CVCL_0022) were cultured in Dulbecco's modified Eagle's medium (DMEM; Welgene, Gyeongsan, Korea) supplemented with 10% fetal bovine serum (FBS) and penicillin–streptomycin. Primary human astrocytes (HAs; ScienCell Research Laboratories, Carlsbad, CA, USA; RRID: CVCL_D585) were maintained in Astrocyte Growth Medium (ScienCell) containing 2% FBS, an astrocyte growth supplement, and penicillin–streptomycin. Human brain microvascular pericytes (HBVPs; ScienCell; RRID: CVCL_0P19) were cultured in Pericyte Medium (ScienCell) supplemented with 2% FBS, a pericyte growth supplement, and penicillin–streptomycin. Human cerebral microvascular endothelial cells (hCMECs/D3; Cedarlane, Canada; RRID: CVCL_0M40) were cultured in Endothelial Growth Medium 2 (EGM2; Lonza Bioscience, Switzerland), containing human epidermal growth factor, hydrocortisone, GA 1000, FBS, vascular endothelial growth factor (VEGF), human fibroblastic growth factor (hFGF‐B), R^3^IGF‐I, ascorbic acid, and heparin (Lonza). Patient‐derived glioblastoma cell lines SNU‐4210 (KCLB No. 04210; established from a glioblastoma of a 79‐year‐old Asian female) and SNU‐5026 (KCLB No. 05026; established from a glioblastoma of a 31‐year‐old Asian male) were obtained from the Korean Cell Line Bank (KCLB, Seoul, Korea) and cultured as monolayers in RPMI‐1640 containing L‐glutamine (300 mg L^−^^1^), 25 mM HEPES and 25 mM NaHCO_3_, supplemented with 10% FBS and penicillin–streptomycin. Both lines are authenticated by short tandem repeat profiling. HAs were used between passages 2 and 7, HBVPs between passages 2 and 10, and hCMEC/D3 cells between passages 27 and 35. U‐87 MG cells and the patient‐derived glioblastoma cell lines were grown in T75 flasks (SPL Life Sciences, Pocheon, Korea), whereas HBVPs and HAs were grown in T75 flasks coated with poly‐L‐lysine, and hCMEC/D3 cells were cultured in T75 flasks coated with rat tail collagen type I solution (C3867, Sigma–Aldrich, St. Louis, MO, USA). Stock solutions of each compound were prepared in 100% DMSO at concentrations adjusted so that the required working concentration was obtained at a final DMSO concentration of 0.5% (v/v) in culture medium. All treatments, including vehicle‐treated controls, therefore contained 0.5% DMSO. The Ru compound was prepared as described previously.

### 5.2 Fabrication of 3D‐Printed Multi‐Well BBB/BBTB Spheroid Culture Platform

To generate BBB and BBTB spheroids, a dual‐mode extrusion‐based 3D bioprinter (INVIVO, ROKIT Healthcare, Seoul, Korea) was employed. Three‐dimensional computer‐aided design (CAD) models for the multi‐microwell chamber and agarose‐coating patterns were constructed using Rhino 6 (Robert McNeel & Associates, Seattle, WA, USA). Each chamber measured 12 × 12 × 4 mm and incorporated nine microwells configured in a 3 × 3 array, with individual microwells measuring 3 × 3 × 4 mm. The CAD files were exported in STL format and imported into the printer control software NewCreatorK (version 1.57.70; ROKIT Healthcare), facilitating direct interaction with the bioprinter. Microwell dishes were fabricated utilizing polylactic acid (PLA) filament extruded at 210°C through a 200‐µm nozzle at a printing speed of 10 mm s^−^^1^ with an infill density of 75%. Immediately after printing, the chambers were sprayed with 70% ethanol and sterilized under UV irradiation in a biosafety cabinet for 30 min.

Agarose coating was achieved using a temperature‐controlled micro‐extrusion system. A 0.6% (w/v) agarose solution prepared in distilled water was loaded into a BD syringe equipped with a Teflon needle (FTN25G, ROKIT Healthcare), wrapped in aluminum foil, and mounted onto the printing cartridge. Agarose was dispensed at 80°C into each microwell, with dimensions of 2.5 × 2.5 × 0.2 mm. Agarose deposition parameters included: nozzle diameter, 0.15 mm; layer height, 0.2 mm; infill density, 100%; filament diameter, 7 mm; filament input flow, 100; dispenser temperature, 80°C; and printing speed, 3 mm s^−^^1^. Immediately after agarose printing, 200 µL of sterile PBS was applied to each well to prevent drying, and was removed immediately before cell printing.

For cell printing, cells were harvested, counted, and resuspended at 2 × 10^5^ cells mL^−^^1^ in BBB medium. BBB medium was prepared from Endothelial Basal Medium 2 (EBM‐2; Lonza Bioscience, Switzerland) supplemented with the EGM‐2 SingleQuots components with the exception of FBS and VEGF, and containing 2% (v/v) human serum (H3667, Merck, Darmstadt, Germany) in place of FBS. VEGF was omitted because VEGF signaling increases endothelial permeability, and human serum was used in place of FBS to better approximate the human vascular environment. BBB spheroids comprised hCMEC/D3 cells, primary human astrocytes and HBVPs mixed at a 1:1:1 ratio; BBTB spheroids comprised hCMEC/D3 cells, U‐87 MG cells and HBVPs at the same ratio. The cell suspension was loaded into a syringe and printed onto the agarose‐coated microwells in a 0.5 × 0.5 × 1 mm pattern, corresponding to 22.5 µL and 4,500 cells per microwell. Cell deposition parameters were as follows: nozzle diameter, 0.2 mm; layer height, 1 mm; infill density, 100%; filament diameter, 5 mm; filament input flow, 100; dispenser temperature, 37°C; and printing speed, 3 mm s^−^^1^. Following printing, spheroids were cultured in BBB medium in a humidified incubator at 37°C with 5% CO_2_. Half of the supernatant was removed daily and replaced with an equal volume of fresh medium, with aspiration performed at the microwell edge to avoid disturbing the forming spheroids. Spheroids formed spontaneously within 24 h and were used for experiments on Day 3, at which point compaction was complete.

### 5.3 Cell Arrangement in BBTB Spheroids

To assess spatial cellular arrangement within BBTB spheroids, U‐87 MG cells, HBVPs, and hCMEC/D3 cells were pre‐labeled with CellTracker Green CMFDA, CellTracker Blue CMAC, and CellTracker CM DiI (all Thermo Fisher Scientific, Waltham, MA, USA), respectively. The cells were incubated with each dye at a 1:1000 dilution for 15 min at 37°C in 5% CO_2_, followed by washing with PBS. Multicellular BBTB spheroids were subsequently formed by co‐culturing labeled HBVPs, hCMEC/D3, and U‐87 MG cells for 72 h as described above. Spheroids were fixed using 4 % paraformaldehyde (PFA), transferred to confocal dishes, and visualized via confocal microscopy (Nanoscope Systems, Daejeon, Korea). Three‐dimensional reconstruction and quantitative evaluation of cell distribution were performed using IMARIS software (Oxford Instruments, UK). To assess whether the layered arrangement is retained, spheroids were cultured under the same conditions until day 5. Each cell population was labeled with a distinct CellTracker dye before assembly. Spheroids were imaged at day 5 using confocal microscopy. The same labeling and imaging procedure was applied to BBTB spheroids assembled with the patient‐derived glioblastoma cell lines SNU‐4210 or SNU‐5026 in place of U‐87 MG cells.

### 5.4 Human Pericyte and U‐87 MG Co‐Culture and Imaging

HBVPs and U‐87 MG cells were co‐seeded at a density of 2 × 10^4^ cells per confocal dish and incubated at 37°C in 5 % CO_2_. Co‐cultures were imaged at 12 and 48 h post‐seeding using a confocal microscope (Nanoscope Systems) to monitor cytoplasmic interactions. Images were analyzed with IMARIS software (Oxford Instruments).

### 5.5 Immunofluorescence Staining of BBTB Spheroids

To examine spatial organization and protein expression, spheroids were collected after 72 h of culture and washed with PBS. For TJ staining (occludin, ZO‐1, and claudin‐5), spheroids were fixed in ice‐cold methanol for 15 min at 4°C. P‐gp staining was performed by fixing spheroids with 4% PFA for 15 min at 25°C, followed by permeabilization with 0.5% (v/v) Tween‐20 for 10 min. After blocking with 2% bovine serum albumin (BSA) and 0.025% Tween‐20 for 30 min, spheroids were incubated overnight at 4°C with primary antibodies against occludin (cat# 33–1500, clone OC‐3F10), ZO‐1 (cat# 33–9100, clone ZO1‐1A12), claudin‐5 (cat# 35–2500, clone 4C3C2), or P‐gp (cat# MA5‐13854, clone F4) (all mouse monoclonal, 1:100; Invitrogen, Carlsbad, CA, USA). After three PBS washes, Alexa Fluor 488‐conjugated donkey anti‐mouse IgG (H+L) secondary antibody (cat# A‐21202; 1:1000; Invitrogen) was applied for 2 h at 25°C. Nuclei were counterstained with Hoechst 33342 (Sigma–Aldrich).

To identify the cell type exhibiting these signals, BBTB spheroids were assembled with hCMEC/D3 cells pre‐labeled with CellTracker Green CMFDA (Thermo Fisher Scientific), leaving HBVPs and U‐87 MG cells unlabeled. The spheroids were immunostained for occludin, ZO‐1, claudin‐5 or P‐gp as described above, using an Alexa Fluor 647‐conjugated donkey anti‐mouse IgG (H+L) secondary antibody (cat# A‐31571; 1:1000; Invitrogen). Using IMARIS, the labeled endothelial cells were reconstructed as a three‐dimensional surface and the protein signals were detected as individual spots, which were then classified by the Colocalize Spots to Surfaces function. Identical settings were applied to all four proteins.

### 5.6 Distribution Assay in Multicellular Spheroids

BBB, BBTB, and U‐87 MG spheroids were generated over 72 h using the previously described 3D printing method. Subsequently, spheroids were incubated with either 10 µg/mL of Dextran– FITC (40 kDa; Sigma‑Aldrich) or the Ru compound (20 µM) in culture medium for 24 h at 37°C in 5% CO_2_. Following incubation, spheroids were washed three times with PBS (5 min each) to remove non‐internalized tracers. The spheroids were then transferred to confocal dishes (SPL Life Sciences) and imaged using a confocal microscope (Nanoscope Systems) under consistent acquisition settings. Regions of interest (ROIs) were defined as cross‐sectional area within the spheroid core at each imaging depth, and depth‐resolved fluorescence intensity profiles were acquired from 8 to 96 µm at 8‐µm intervals. Mean fluorescence intensity (MFI) at each depth within the spheroid core was quantified using MetaMorph software (Molecular Devices) and further analyzed in Microsoft Excel (Microsoft, Redmond, WA, USA). Each ratio was obtained within a single probe, so the difference in molecular weight between Dextran‐FITC and the Ru compound does not contribute to the comparison. Comparisons of intensity between spheroid types were made at the single depth of 72 µm.

### 5.7 Intracellular Localization of the Ru Compound

U‐87 MG cells were seeded on confocal dishes (SPL Life Sciences) at a density of 2 × 10^4^ cells per dish and incubated for 24 h at 37°C in 5% CO_2_. Subsequently, cells were treated with 10 µM Ru compound in DMEM for 2 h at 37°C. Post‐treatment, cells were washed with PBS and incubated with either MitoTracker Red (100 nM; Invitrogen) or ER‐Tracker Red (100 nM; Invitrogen) in DMEM to selectively label mitochondria and endoplasmic reticulum (ER), respectively. Cells were then fixed with 4% PFA for 15 min and counterstained with Hoechst 33342 (Sigma–Aldrich) to visualize nuclei. Subcellular localization of the Ru compound was assessed using confocal microscopy (Nanoscope Systems) and IMARIS software (Oxford Instruments). To assess crosstalk between the Ru and trackers, control spectra of the Ruthenium compound alone and the mitochondrial/ER trackers alone were obtained. U‐87 MG cells were treated with the Ru compound alone (10 µM, 2 h), or with ER‐Tracker Red alone (100 nM, 30 min), or with MitoTracker Red alone (100 nM, 30 min). Two emission wavelength regions were obtained with different excitation wavelengths: Ex 488 nm / Em 593/46 nm, and Ex 561 nm / Em 620/14 nm (Figure S3).

### 5.8 Intracellular ROS Assay

U‐87 MG cells were seeded on confocal dishes and incubated overnight at 37°C in 5 % CO_2_. Cells were then incubated in DMEM with or without the Ru compound (5, 10, or 20 µM) for 6 h. After washing with PBS, cells were treated with 20 µM 7′‐dichlorofluorescin diacetate (DCFDA) for 15 min at 25°C in the dark. After an additional PBS wash, intracellular ROS‐dependent DCFDA fluorescence was visualized via confocal microscopy (Nanoscope Systems) and quantified with MetaMorph software (Molecular Devices).

### 5.9 Intracellular MPT Assay

U‐87 MG cells were seeded on confocal dishes and incubated overnight at 37°C in 5% CO_2_. Cells were then treated with or without the Ru compound (5, 10, or 20 µM) in DMEM for 48 h. After washing with PBS, cells were incubated with 2 µM calcein acetoxymethyl ester (Calcein AM; Invitrogen) for 30 min at 37°C in 5 % CO_2_, followed by the addition of CoCl_2_ and further incubation for 10 min in the dark. After a final PBS wash, mitochondrial permeability transition (MPT)‐dependent calcein fluorescence was imaged using a confocal microscope (Nanoscope Systems) and analyzed with MetaMorph software (Molecular Devices).

### 5.10 Intracellular Ca^2^^+^ Assay

U‐87 MG cells were seeded on confocal dishes and incubated overnight at 37°C in 5% CO_2_. Cells were then treated with or without the Ru compound (5, 10, or 20 µM) in DMEM for 48 h. After washing with PBS, cells were incubated with 3 µM Indo‐1 AM (Invitrogen) for 30 min at 37°C in 5% CO_2_ in the dark. Cells were washed with PBS, and Indo‐1 fluorescence—reflecting intracellular Ca^2^^+^ levels—was visualized by confocal microscopy (Nanoscope Systems) and quantified using MetaMorph software (Molecular Devices).

### 5.11 In Vitro Cytotoxicity Assay of the Ru Compound

The cytotoxic effects of the Ru compound, temozolomide (TMZ), osimertinib, paclitaxel (PTX), and cisplatin (CIS) against U‐87 MG cells were evaluated by the 3‐(4,5‐dimethylthiazol‐2‐yl)‐2,5‐diphenyltetrazolium bromide (MTT) assay to determine IC_50_ values for subsequent BBTB spheroid studies. Briefly, 1 × 10^4^ cells per well were seeded in 96‐well plates and cultured for 24 h at 37°C in 5% CO_2_. Cells were then treated with serial dilutions of each compound in DMEM for 48 h at the following concentration ranges: Ru compound (0–100 µM), TMZ (0–1000 µM), osimertinib (0–100 µM), PTX (0–50 µM), and CIS (0–100 µM). Post‐treatment, the medium was replaced with DMEM containing 0.5 mg/mL MTT (Invitrogen), and the cells were incubated for 2 h in the dark at 37°C. The MTT solution was removed, and 100 µL of dimethyl sulfoxide (DMSO) was added to each well to dissolve the formazan crystals. Absorbance was measured at 570 nm with a microplate reader (Molecular Devices). Cell viability was expressed as a percentage relative to vehicle‐treated controls, and IC_50_ values were determined by nonlinear regression analysis using GraphPad Prism software.

### 5.12 Immunofluorescence Analysis of α‐Smooth Muscle Actin (α‐SMA) and F‐Actin in Pericytes

To assess the phenotypic characteristics of pericytes, HBVPs were seeded at a density of 1 × 10^5^ cells per confocal dish (SPL Life Sciences) pre‐coated with rat tail collagen type I. The cells were then treated with or without the Ru compound (10 µM) in Pericyte Medium. After 48 h treatment, cells were fixed with 4% PFA for 15 min and permeabilized with 0.5% (v/v) Tween‐20 in PBS for 10 min. After blocking with 2% BSA for 30 min at 25°C, the cells were incubated overnight at 4°C with a primary antibody against α‐SMA (cat# MA1‐06110, clone 1A4;1:100; Invitrogen) diluted in 2% BSA. After three washes with PBS, cells were incubated with Alexa Fluor 488‐conjugated donkey anti‐mouse IgG (H+L) secondary antibody (cat# A‐21202;1:1000; Invitrogen) for 2 h at 25°C.

To visualize filamentous actin (F‐actin), cells were stained with Phalloidin‐iFluor 647 (cat# ab176759;1:1000;Abcam, Cambridge, UK) for 20 min. Nuclei were counterstained with Hoechst 33342 (Sigma–Aldrich). Fluorescence images were acquired using a confocal microscope (Nanoscope Systems), which were analyzed with MetaMorph software (Molecular Devices).

### 5.13 Immunofluorescence Analysis of MMP‐9

To assess MMP‐9 expression, hCMEC/D3 cells and HAs were seeded at a density of 1 × 10^5^ cells per confocal dish (SPL Life Sciences) pre‐coated with rat tail collagen type I. Cells were then treated with the Ru compound in the respective growth media. Post‐treatment, cells were fixed with 4% PFA for 15 min and permeabilized with 0.5% (v/v) Tween‐20 in PBS for 10 min. After blocking with 2% BSA in PBS for 30 min at RT, the cells were incubated overnight at 4°C with a rabbit anti‐MMP‐9 primary antibody (cat# MA5‐32705;1:100; Invitrogen) diluted in 2% BSA. After three washes with PBS, cells were incubated with Alexa Fluor 594‐conjugated donkey anti‐rabbit IgG (cat# A‐21207;1:1000; Invitrogen) for 2 h at 25°C. Nuclei were counterstained with Hoechst 33342 (Sigma–Aldrich). Fluorescence images were acquired with a confocal microscope (Nanoscope Systems) and analyzed using MetaMorph software (Molecular Devices).

### 5.14 Expressions of MMP‐9 and Endothelin‐1 Using Enzyme‐Linked Immunosorbent Assay (ELISA)

The secretion levels of MMP‐9 and ET‐1 from hCMEC/D3 cells and HAs were quantified using MMP‐9 and ET‐1 ELISA kits (Thermo Fisher Scientific) according to the manufacturer's instructions. Cells were cultured to confluence in 6‐well plates and maintained in their respective media with or without the Ru compound for 48 h. Cell culture supernatants were collected, centrifuged at 12 000 × g for 10 min at 4°C, and the clarified supernatants were evaluated via ELISAs.

### 5.15 Immunofluorescence Analysis of Occludin and ZO‐1 in 2D hCMEC/D3 Cultures

hCMEC/D3 cells were grown to confluence on collagen type I‐coated confocal dishes (SPL Life Sciences) and fixed with methanol for 15 min at 4°C. Fixed cells were blocked with 2% BSA in PBS and incubated overnight at 4°C with mouse anti‐occludin (cat# 33–1500, clone OC‐3F10; 1:100; Invitrogen) and mouse anti‐ZO‐1 (cat# 33–9100, clone ZO1‐1A12; 1:100; Invitrogen) primary antibodies. After three PBS washes, cells were incubated with Alexa Fluor 488 donkey anti‐mouse IgG (cat# A‐21202; 1:1000;Invitrogen) for 2 h at 25°C. Nuclei were counterstained with Hoechst 33342, and images were acquired using a confocal microscope (Nanoscope Systems) and analyzed with MetaMorph software (Molecular Devices).

### 5.16 Western Blot Analysis of Occludin and ZO‐1 in hCMEC/D3 Cells

hCMEC/D3 cells were cultured to approximately 95% confluence and treated with the Ru compound (10 and 20 µM) for 48 h. For the ROS scavenger experiment, cells were pretreated with N‐acetylcysteine as described above before Ru exposure. Post‐treatment, cells were lysed in radioimmunoprecipitation assay (RIPA) buffer supplemented with a 1% protease inhibitor cocktail. Total protein concentrations were quantified using a BCA protein assay kit. Equal amounts of protein (40 µg per lane) were separated by 10% sodium dodecyl sulfate–polyacrylamide gel electrophoresis (SDS‐PAGE) at 100 V for 90 min, then transferred onto polyvinylidene fluoride (PVDF) membranes. Membranes were blocked with 5% BSA in TBST to prevent non‐specific binding before overnight incubation at 4°C with primary antibodies against ZO‐1 (cat# 61–7300;1:1000; Thermo Fisher Scientific), occludin (cat# sc‐133256;1:250; Santa Cruz Biotechnology, Dallas, TX, USA), and GAPDH (cat# MA5‐15738;1:5000; Thermo Fisher Scientific). Following three washes with TBST, the membranes were incubated for 1 h at 25 °C with HRP‐conjugated goat anti‐rabbit (1:10 000) or anti‐mouse IgG (1:5000; Thermo Fisher Scientific) secondary antibodies. Protein bands were detected using an enhanced chemiluminescence (ECL) substrate (Thermo Fisher Scientific) and imaged with the ImageQuant 800 system (Cytiva, Marlborough, MA, USA). Membranes were stripped and re‐probed to detect the remaining targets, with GAPDH detected on the same membrane as a loading control for each protein. Band intensities were quantified using ImageJ (version 1.54g; NIH, Bethesda, MD, USA), normalized to the GAPDH signal in the corresponding lane, and expressed relative to the vehicle‐treated control within each independent experiment.

### 5.17 Assessment of Anticancer Effects in BBTB Spheroids Using 7‐AAD Staining

Multicellular BBTB spheroids were generated over 72 h as described above. These spheroids were treated with the Ru compound (20 µM), TMZ (240 µM), PTX (5 µM), and CIS (9 µM) for 48 h at 37°C in 5% CO_2_. Vehicle‐treated spheroids served as controls. After treatment, the spheroids were washed three times with PBS and incubated for 1 h in a 7‐AAD solution (1:20 dilution) at 37°C in 5% CO_2_. After three additional PBS washes, the spheroids were transferred to confocal dishes and imaged with a confocal microscope (Nanoscope Systems) under identical acquisition settings. Depth‐resolved fluorescence intensity profiles were quantified using MetaMorph software (Molecular Devices). The same procedure was applied to BBTB spheroids assembled with the patient‐derived glioblastoma cell lines SNU‐4210 and SNU‐5026 in place of U‐87 MG cells, which were treated with the Ru compound (20 µM) or TMZ (240 µM) for 48 h.

To determine the cellular origin of the 7‐AAD signal, BBTB spheroids were assembled with U‐87 MG cells pre‐labeled with CellTracker Green CMFDA (Thermo Fisher Scientific) as described above, with hCMEC/D3 cells and HBVPs left unlabeled. At day 3, spheroids were treated with the Ru compound (20 µM) for 48 h, stained with 7‐AAD as described, and counterstained with Hoechst 33342. CellTracker Green CMFDA was excited at 488 nm and its emission was collected at 525/50 nm, 7‐AAD was excited at 561 nm and its emission was collected above 640 nm, and Hoechst 33342 was excited at 405 nm and its emission was collected at 447/60 nm. Using IMARIS (Oxford Instruments), the labeled tumor population was reconstructed as a three‐dimensional volume and 7‐AAD‐positive cells were detected as spots, which were then classified by the Colocalize Spots to Surfaces function.

### 5.18 ROS Scavenger Assay

hCMEC/D3 cells and primary human astrocytes were cultured to confluence and assigned to four groups: vehicle‐treated control, NAC alone, Ru alone, and NAC pretreatment followed by Ru exposure. For the pretreatment groups, cells were incubated with N‐acetylcysteine (5 mM) for 2 h, after which the NAC‐containing medium was removed and the cells were washed with PBS before exposure to the Ru compound (20 µM) for 48 h. Culture supernatants were collected and centrifuged at 12 000 × g for 10 min at 4°C, and the clarified supernatants were analyzed using a human endothelin‐1 ELISA kit (EIAET1, Invitrogen, Thermo Fisher Scientific) and a human MMP‐9 ELISA kit (BMS2016‐2, Invitrogen, Thermo Fisher Scientific) according to the manufacturers' instructions. Concentrations were interpolated from a four‐parameter logistic standard curve and corrected for the sample dilution. Suppression of the Ru‐induced increase was calculated as the difference between the Ru and NAC‐pretreated groups expressed as a percentage of the difference between the Ru and control groups. In parallel, cells were processed for immunofluorescence analysis of ZO‐1 and occludin and for immunoblotting as described in the preceding section.

### 5.19 Cell Viability Assay

To establish appropriate drug concentrations for Transwell permeability testing, cytotoxicity was assessed. hCMEC/D3 cells were seeded at a density of 1 × 10^4^ cells per well in 96‐well white‐walled clear‐bottom plates (#11515, SPL Life Sciences) pre‐coated with 0.1 mg/mL rat tail collagen type I (C3867, Sigma–Aldrich; in 0.02 N acetic acid) and cultured for 2 days. Cells were treated with various concentrations of the test compounds for 24 h. Cell viability was quantified using the CellTiter‐Glo Luminescent Cell Viability Assay (Promega, USA), and luminescence was detected with a multi‐mode microplate reader (iD3, No.045‐1, Molecular Devices). Drug concentrations that maintained at least 90% cell viability relative to controls were selected for subsequent studies.

### 5.20 Transwell Permeability Assay

Cell culture. SPLinsert 24‐well Transwell inserts (0.4 µm pore, #35024, SPL Life Sciences) were coated with rat tail collagen type I (0.1 mg/mL) for 1 h and washed with PBS. hCMEC/D3 cells were seeded at 2 × 10^5^ cells/cm^2^ with 200 µL in the apical chamber and 800 µL in the basolateral chamber, and cultured for 7 days at 37°C under 5% CO_2_. The medium was changed every 2–3 days.

Monolayer integrity assessment. After washing with transport buffer (HBSS containing 25 mM HEPES, 25 mM glucose, 350 mg/L NaHCO_3_, pH 7.4), cells were equilibrated for 30 min. Lucifer Yellow (100 µM, L0259, Sigma–Aldrich) in 200 µL transport buffer was added to the apical chamber, while 800 µL buffer was added to the basolateral chamber. Following a 60‐min incubation at 37°C with agitation (100 rpm), basolateral fluorescence (Ex/Em 485/530 nm, iD3, Molecular Devices) was measured and compared with blank inserts to confirm barrier integrity.

P‐gp functional validation. P‐gp activity was assessed with Rhodamine 123 (5 µM, R8004, Sigma–Aldrich) in the presence or absence of a P‐gp inhibitor, verapamil (100 µM, V4629, Sigma–Aldrich). Cells were pre‐incubated with verapamil for 30 min, which was retained in the donor solution throughout the assay. During the 60‐min incubation, transwell inserts were sequentially transferred to fresh basolateral wells at 15, 30, 45, and 60 min, and the basolateral solutions remaining in the previous wells were collected. Fluorescence was then measured to calculate the apparent permeability (P_app_) using the following equation:

$$P_{app}\left(cm/s\right)=\left(dQ/dt\right)/\left(A\times C_{0}\right)$$

where *dQ/dt* is the permeation rate (pmol/s), *A* is the membrane area (0.33 cm^2^), and *C_0_* is the initial donor concentration (µM).

Drug permeability. After equilibration in transport buffer, test compounds—Ru compound (20 µM), TMZ (240 µM), and osimertinib (0.5 µM)—were added to the apical chamber. For P‐gp inhibition experiments, cells were pre‐treated and co‐treated with verapamil (100 µM). During incubation at 37°C with shaking (100 rpm), inserts were transferred to fresh basolateral wells at 15, 30, 45, and 60 min to measure permeation over time. Samples were stored at −80°C until analysis. TMZ and osimertinib were quantified by liquid chromatography tandem mass spectrometry (LC–MS/MS; TSQ Altis/Quantis, Thermo Fisher Scientific), while Ru compound, Rhodamine 123, and Lucifer Yellow were analyzed via fluorescence spectrophotometry.

Sample preparation for LC‐MS/MS. Basolateral samples were processed using analyte‐specific extraction methods. For osimertinib, samples were combined with cold acetonitrile containing 0.1% formic acid at a 1:1 (v/v) ratio. For TMZ, the extraction solvent comprised 0.1% formic acid in water and 0.1% formic acid in acetonitrile (1:1, v/v), mixed with the samples at a 1:4 volume ratio. After vortexing, both mixtures were centrifuged at 14 000 × g for 10 min at 4°C, and supernatants were transferred to 2‐mL autosampler vials. Drug‐free blanks were prepared alongside samples to identify and eliminate background ion interference.

LC‐MS/MS analytical conditions. Osimertinib and TMZ were quantified using individually optimized LC–MS/MS methods on a Thermo Scientific Vanquish UHPLC coupled to a TSQ Altis triple quadrupole mass spectrometer with an electrospray ionization (ESI) source. Chromatography employed a Hypersil GOLD column (2.1 × 100 mm, 1.9 µm, C18) maintained at 30°C. Common ESI parameters included: spray voltage, 3,500 V (positive mode); sheath gas, 45 Arb; auxiliary gas, 10 Arb; ion transfer tube, 325°C; vaporizer, 350°C. Osimertinib analysis involved gradient elution: 0–0.5 min, 15% B; 0.5–3.5 min, 15%–95% B; 3.5–8.0 min, 95% B; 8.0–8.6 min, 95%–15% B; 8.6–10.0 min, 15% B; flow rate 0.5 mL/min; injection 2 µL. SRM quantifier transition: *m/z* 500.267 → 71.984 (CE 25.93 eV); qualifiers: *m/z* 500.267 → 385.067 (CE 36.95 eV) and *m/z* 500.267 → 455.097 (CE 23.3 eV); RF lens set at 124 V. The TMZ analysis used isocratic elution at 60% B (flow rate 0.2 mL/min; injection volume 2 µL). SRM quantifier transition: *m/z* 194.850 → 137.933 (CE 9.55 eV); qualifiers: *m/z* 194.850 → 54.933 (CE 22.79 eV) and *m/z* 194.850 → 66.934 (CE 19.61 eV); RF lens at 32 V. Each transition had a dwell time of 199.001 ms, with Q1/Q3 resolution at 0.7 FWHM for both analytes.

LC‐MS/MS data processing and quantification. Data acquisition and quantitative analysis were performed using Thermo Xcalibur software (v4.7.69.39) through the QuanBrowser module. Peaks were integrated using the ICIS algorithm, and quantification was achieved with external standard calibration based on absolute peak areas. Calibration curves spanned 1–80 nM for osimertinib (quadratic regression, 1/x_2_ weighting, origin ignored) and 100–20,000 nM for TMZ (linear regression, 1/x_2_ weighting, origin ignored).

### 5.21 Analysis of Cell Death via Flow Cytometry

U‐87 MG cells were stained with CellTracker Green CMFDA (Thermo Fisher Scientific). Multicellular BBTB spheroids were formed over 72 h as previously described. The BBTB spheroids were subsequently treated with the Ru compound, CIS, PTX, or TMZ at their IC_50_ concentrations for U‐87 MG cells and incubated for 48 h at 37°C in 5% CO_2_. Post‐incubation, spheroids were washed with PBS and trypsinized into single cells. These single cells were then incubated with 7‐AAD solution (1:20) for 1 h at 37°C in 5% CO_2_. After washing with PBS, cells were resuspended in FACS buffer for flow cytometric analysis, which was performed on a BD flow cytometer to enable quantitative evaluation.

### 5.22 Statistical Analysis

#### 5.22.1 Pre‐Processing of Data

No data transformation was applied. Immunofluorescence intensities were background‐corrected against a cell‐free field acquired under identical settings and are reported in arbitrary units (a.u.). Cell‐viability values were normalized to the vehicle‐treated control of the same cell type and are expressed as a percentage of that control. Immunoblot band intensities were normalized to the GAPDH signal in the corresponding lane and expressed relative to the vehicle‐treated control within each independent experiment. ELISA concentrations were interpolated from a four‐parameter logistic standard curve and corrected for sample dilution. No outliers were identified or excluded, and no data points were removed from any analysis.

#### 5.22.2 Data Presentation

All data are presented as mean ± standard deviation (SD). Standard error of the mean is not used anywhere in this manuscript or in the Supporting Information.

#### 5.22.3 Sample Size

The unit to which n refers, and the number of measurements made per replicate, are stated in the corresponding figure legend for every panel. For monolayer experiments, n is the number of confocal dishes, culture wells or Transwell inserts that were seeded and treated in parallel. For spheroid experiments, n is the number of individual spheroids, each formed in a separate microwell and treated separately.

#### 5.22.4 Statistical Methods

Comparisons among three or more groups were made by ordinary one‐way ANOVA followed by Tukey's multiple‐comparisons test. Comparisons between two groups were made by an unpaired two‐tailed Student's t‐test. All tests were two‐sided with a testing level (alpha) of 0.05. Significance is indicated as p < 0.05 (*), p < 0.01 (**), p < 0.001 (***) and p < 0.0001 (****); n.s. denotes not significant.

#### 5.22.5 Software

All statistical analyses and graph preparation were performed in GraphPad Prism 11.0.2 (GraphPad Software, Boston, MA, USA). Image quantification was performed in MetaMorph (Molecular Devices), IMARIS (Oxford Instruments) and ImageJ (NIH), as specified in the corresponding subsections above.

#### 5.22.6 Copyright Statement

All figures and the Table of Contents graphic were prepared by the authors for this work, and no previously published figure has been reprinted or adapted. Figure 1 and the Table of Contents graphic were assembled from BioRender library elements. The corresponding BioRender citation links are provided in the Figure 1 legend and the Table of Contents graphic caption.

## Author Contributions

**Hyunjung Lee**: conceptualization, investigation, methodology, visualization, formal analysis, writing – original draft, writing – review and editing. **Chan Ho Jeon**: conceptualization, investigation, methodology, formal analysis, writing – original draft, visualization. **Doyun Kim**: investigation. **Minjae Lee**: investigation. **Minji Kwon**: investigation. **Joon Myong Song**: conceptualization, funding acquisition, supervision, writing – review and editing, project administration, writing – original draft, resources.

## Funding

This research was funded by grants from the National Research Foundation of Korea (NRF), supported by the Ministry of Education, Science, and Technology (MEST) under Project Numbers 2023R1A2C3006991 and RS‐2023‐00218543.

## Conflicts of Interest

The authors declare no conflicts of interest.

## Supporting information




**Supporting File**: advs78150‐sup‐0001‐SuppMat.docx.

## Data Availability

The datasets used and/or analyzed during the current study are available from the corresponding author on reasonable request.
