# Supplementary material for: 3D Bioprinted Blood–Brain–Tumor Barrier Spheroid‐Based In Situ Evaluation of Anticancer Compounds Against Glioblastoma: ROS‐Producing Ruthenium as a Potential Therapeutic Candidate
Source: Adv Sci (Weinh). 2026 Oct 6:e78150. Online ahead of print. doi: 10.1002/advs.78150 (PMC13643098; doi:10.1002/advs.78150)
Supplement: Supplementary file 1 [file ADVS-9999-e78150-s001.docx]

**Advanced Science**

**Supporting Information**

**3D Bioprinted Blood–Brain–Tumor Barrier Spheroid-based In Situ Evaluation of Anticancer Compounds Against Glioblastoma:**

**ROS-Producing Ruthenium as a Potential Therapeutic Candidate**

Hyunjung Lee^[a]+^, Chan Ho Jeon^[a]+^, Doyun Kim^[a]^, Minjae Lee^[a]^, Minji Kwon^[a]^ and Joon Myong Song ^*[a]^

[a] College of Pharmacy, Seoul National University, Seoul 08826, Republic of Korea

+ These authors contributed equally to this work.

* Corresponding author : jmsong@snu.ac.kr (J. M. Song)

**Table of Contents**

**Figure S1.** Cellular arrangement of BBTB spheroids at day 5	3

**Figure S2.** Cell type exhibiting BBB-associated protein signals in BBTB spheroids	4

**Figure S3.** Single-label controls for the subcellular localization assay	5

**Figure S4.** Uncropped immunoblot images	8

**Figure S5.** Cell type showing the 7-AAD signal in drug-treated BBTB spheroids.	9

**Figure S6.** Cellular arrangement of BBTB spheroids assembled with patient-derived glioblastoma cell lines	11

**Figure S7.** Paracellular permeability of the hCMEC/D3 monolayer assessed using Lucifer Yellow	12

**Figure S1. Cellular arrangement of BBTB spheroids at day 5.**

Spheroids were assembled with each cell population labeled with a distinct CellTracker™ dye as Figure 2 and were cultured under the same conditions until day 5. Panels show U-87 MG cells (green) with hCMEC/D3 cells (red) **(A)**, with HBVPs (blue) **(B)**, and all three cell types **(C)**. The hCMEC/D3 cells formed the outermost layer, the HBVPs occupied an intermediate position and the U-87 MG cells formed the core. CellTracker™ signal is diluted by cell division, so intensities at day 5 are not comparable with those at day 3 and do not indicate population size. Each panel shows a representative spheroid. Spheroids from a single preparation (n = 3) were examined with consistent results. No statistical comparison was performed. Scale bar = 100 µm.

**Figure S2. Cell type exhibiting BBB-associated protein signals in BBTB spheroids.**

**(A–D)** BBTB spheroids containing CellTracker™ Green CMFDA-labeled hCMEC/D3 cells were immunostained for P-gp **(A)**, ZO-1 **(B)**, claudin-5 **(C)** or occludin **(D)** (shown in red). Columns show the Hoechst 33342 nuclear staining **(1)**, the labeled endothelial cells **(2)**, the target protein **(3)**, the overlay of the endothelial label and the protein signal **(4)**, and the full overlay **(5)**. Images are three-dimensional views of confocal z-stacks. **(E)** Representative surface and spot analysis used for quantification, shown here for ZO-1. Protein signals colocalized with the endothelial surface are shown in yellow and non-colocalized signals in red. The boxed region is enlarged at right. **(F)** Proportion of protein signals located within the endothelial surface. Data are presented as mean ± SD of n = 3 individual spheroids per group from a single independent experiment. No statistical comparison was performed between proteins. Scale bars = 100 µm **(A–D, E left)** and 40 µm **(E right)**.

**Figure S3. Single-label controls for the subcellular localization assay.**

U-87 MG cells were treated with the Ru compound alone (upper row), or ER-Tracker™ Red alone (middle row), or MitoTracker™ Red alone (lower row). Each spectrum was acquired under the identical condition used for Figure 6A and 6B. The Ru compound was detected at Ex 488 nm and Em 593/46 nm (green). The organelle trackers were detected at Ex 561 nm and Em 620/14 nm (red). Hoechst 33342 was detected at Ex 405 nm and Em 447/60 nm (blue). Emission from the Ru compound appeared only in the 593/46 nm channel, and emission from both trackers appeared only in the 620/14 nm channel. Images are representative of three confocal dishes seeded in parallel per condition (n = 3). No statistical comparison was performed. Scale bar = 20 µm.

**Figure S4. Uncropped immunoblot images.**

Full, uncropped chemiluminescence images of the membranes from which the cropped panels in Figure 8C and Figure 9G were prepared. Images are shown in the following order: occludin and ZO-1 for Figure 8C, followed by occludin for Figure 9G and ZO-1 for Figure 9G. Each target protein and its GAPDH loading control were detected on the same membrane and in the same lanes, with the membrane stripped after detection of the target protein and re-probed for GAPDH. Molecular weight markers are indicated at left, and the expected position of each target is marked with an arrowhead. Magenta denotes pixels at or above the saturation limit of the detector. Where the GAPDH signal was saturated at the exposure used for the target protein, a separate image of the same membrane acquired at a shorter exposure is shown beneath the target protein image, and all band intensities were quantified from these non-saturated images. The blots for Figure 8C were resolved with three lanes per condition from three biological replicates; the blots for Figure 9G were obtained from a single experiment with one lane per condition.

**Figure S5. Cell type showing the 7-AAD signal in drug-treated BBTB spheroids.**

BBTB spheroids were assembled with U-87 MG cells pre-labeled with CellTracker™ Green CMFDA, with hCMEC/D3 cells and HBVPs left unlabeled. At day 3, spheroids were treated with the Ru compound (20 µM) for 48 h and stained with 7-AAD. **(A)** Three-dimensional rendering showing the CellTracker-labeled tumor population (green) and 7-AAD signal (red). **(B)** The same spheroid with Hoechst 33342 nuclear counterstain (blue), showing the unlabeled cells surrounding the labeled tumor core. **(C)** Optical sections at the indicated depths from the spheroid surface. **(D)** Orthogonal sections through the spheroid, with xz and yz views shown at right and below. Panels **(A, B)**, **(C)** and **(D)** show separate spheroids. 91 ± 5.45% of 7-AAD-positive objects were located within the CellTracker-labeled tumor volume (n = 3 separately formed spheroids from a single independent experiment). Each panel shows a representative spheroid. Scale bars = 100 µm.

**Figure S6. Cellular arrangement of BBTB spheroids assembled with patient-derived glioblastoma cell lines.**

BBTB spheroids were assembled with SNU-4210 **(A)** or SNU-5026 **(B)** in place of U-87 MG cells, together with hCMEC/D3 endothelial cells and HBVPs at a 1:1:1 ratio. Each cell type was labeled with a distinct CellTracker™ dye prior to spheroid assembly, and day-3 spheroids were imaged by confocal microscopy. In both constructs, hCMEC/D3 cells occupied the outermost layer, HBVPs formed an intermediate layer, and the patient-derived glioblastoma cells comprised the core, matching the organization observed for the U-87 MG-based model **(Figure 2B–D)**. Each panel shows one representative spheroid; three separately formed spheroids from a single preparation were examined per cell line, with consistent results. Scale bars = 70 µm.

**Figure S7. Paracellular permeability of the hCMEC/D3 monolayer assessed using Lucifer Yellow.**

Paracellular permeability of the hCMEC/D3 monolayer assessed using Lucifer Yellow (LY). The apparent permeability coefficient (P_app_) of LY across the hCMEC/D3 monolayer is presented to confirm monolayer integrity prior to drug transport assays. Data are presented as mean ± SD of n = 3 Transwell inserts seeded in parallel.
